# Global, regional, and national burden of intracerebral hemorrhage and its attributable risk factors from 1990 to 2021: results from the 2021 Global Burden of Disease Study

**DOI:** 10.1186/s12889-024-19923-7

**Published:** 2024-09-06

**Authors:** Libo Xu, Zhenhao Wang, Wenchao Wu, Mao Li, Qingsong Li

**Affiliations:** 1 The Second Affiliated Hospital of Harbin Medical University, Harbin, 150086 China; 2 University of California, Davis, California 95616 USA

**Keywords:** Intracerebral hemorrhage, Global Burden of Disease, Sociodemographic index, Risk factor analysis, Joinpoint

## Abstract

**Background:**

Intracerebral hemorrhage (ICH) results from the rupture of blood vessels causing bleeding within the brain and is one of the major causes of death and long-term disability globally, particularly in low- and middle-income countries. Despite having a lower incidence than ischemic stroke, ICH imposes a greater social and economic burden. To our knowledge, since the release of the 2021 Global Burden of Disease (GBD) report, there has been no comprehensive update on the epidemiology and trends of ICH. This study aims to analyze the impact of gender, age, and the Sociodemographic Index (SDI) on the burden of ICH at global, regional, and national levels.

**Methods:**

Data on the incidence, deaths, and disability-adjusted life years (DALYs) of ICH and its related risk factors from 1990 to 2021 were extracted from the GBD 2021 project, encompassing 203 countries and regions. Furthermore, temporal trends of the global intracerebral hemorrhage burden were assessed through Joinpoint analysis.

**Results:**

In 2021, there were 3.444 million new cases of ICH worldwide, with an age-standardized prevalence rate of 40.8 per 100,000 people, representing a 31.4% decrease compared to 1990. In 2021, ICH caused 3.308 million deaths, with an age-standardized mortality rate of 39.1 per 100,000 people, a reduction of 36.6% since 1990. Globally, ICH accounted for 79.457 million DALYs, with an age-standardized DALY rate of 92.4 per 100,000 people, representing a 39.1% decrease since 1990. Regionally, Central Asia, Oceania, and Southeast Asia had the highest age-standardized prevalence rates of ICH, whereas Australasia, high-income North America, and Western Europe had the lowest rates. Nationally, the Solomon Islands, Mongolia, and Kiribati had the highest age-standardized prevalence rates, whereas Switzerland, New Zealand, and Australia had the lowest. Hypertension, smoking, and environmental pollution were identified as the primary risk factors for ICH. This study also validated the significant association between SDI and the burden of ICH, with the age-standardized DALY rate of ICH decreasing significantly as SDI increased.

**Conclusion:**

Despite the decreasing burden of intracerebral hemorrhage, it remains a significant public health issue in countries with a lower SDI. Prevention strategies should prioritize hypertension management, air quality improvement, and smoking control to further mitigate the impact of intracerebral hemorrhage.

**Supplementary Information:**

The online version contains supplementary material available at 10.1186/s12889-024-19923-7.

## Introduction

Intracerebral hemorrhage (ICH) is a leading cause of death and long-term disability among adults globally, particularly in low- and middle-income countries, making it a major public health issue [1–3]. Studies show that although the incidence of hemorrhagic stroke is lower than that of ischemic stroke, ICH imposes a greater social and economic burden [4, 5].

The Global Burden of Disease (GBD) study provides detailed epidemiological data, offering critical insights for public health decision-making through systematic analysis of various diseases and risk factors [6, 7]. The GBD database includes comprehensive data from 1990 to 2021, covering incidence, mortality, and disability-adjusted life years (DALYs), invaluable for assessing global trends and influencing factors of ICH [8, 9].

Using data from GBD 2021, we conducted a comprehensive assessment of the global, regional, and national burden of ICH and its associated risk factors. This study aims to analyze the impact of gender, age, and the SDI on the burden of ICH at global, regional, and national levels. Although previous research has provided data up to 2019, the rapidly evolving field of stroke research necessitates the latest two years of data to remain relevant for public health policymakers. Additionally, since the onset of the COVID-19 pandemic in late 2019, healthcare resources have been predominantly allocated to controlling and treating the pandemic. This period from 2019 to 2021 underscores the critical importance of providing updated data. Therefore, this study updates the data to 2021, offering the latest epidemiological trends and risk factor analysis to support timely prevention and intervention measures.

## Methods

### Overview

This study analyzes the epidemiology of ICH using annual incidence, mortality, and DALY data from the GBD database. This study strictly adheres to the Guidelines for Accurate Health Estimates Reporting recommendation [10].

### Estimation framework

The incidence of ICH is estimated using DisMod-MR, a Bayesian meta-regression modeling tool [11]. Adjusted available mortality data are used to estimate the mortality rate of ICH, employing a cause-of-death ensemble model.

### Data availability

The data for this study were obtained from the GBD 2021 database, covering data from 1990 to 2021. The definition of ICH is based on the International Classification of Diseases, 10th Revision (ICD-10) standards. All research data can be accessed publicly via the Global Health Data Exchange (GHDx) query tool (https://vizhub.healthdata.org/gbd-results/).

### Patient and public involvement

Since the study utilized publicly available aggregate data, no patients were involved in setting the research question, determining the outcome measures, or participating in the design or implementation of the study.

### Statistical analyses

To estimate the years of life lost, the number of deaths in each age group was multiplied by the remaining life expectancy for that age group according to the Global Burden of Disease standard life table. The DALYs for intracerebral hemorrhage were calculated by summing the years lived with disability (YLD) and the years of life lost (YLL) [11, 12]. The level of uncertainty was assessed by performing 1,000 iterations at each step and integrating uncertainties from multiple sources, including input data, measurement error corrections, and residual non-sampling error estimates. The uncertainty interval was defined as the range between the 25th and 975th values of the ordered samples. A smoothed spline model was employed to analyze the relationship between the burden of ICH and the SDI across 21 regions and 203 countries and territories. The Sociodemographic Index ranges from 0 (least developed) to 1 (most developed) and is a composite measure including GDP per capita (smoothed over the past decade), average years of education for those aged 15 and older, and total fertility rate for those under 25 years old [13, 14]. The temporal trends in the global burden of intracerebral hemorrhage were estimated using the Joinpoint regression model, a statistical linear model set previously detailed in the literature [15]. Annual percentage changes (APCs) and their 95% confidence intervals (CIs) were calculated. Age-standardized incidence rates, mortality rates, and DALY rates were plotted using R software (version 4.3.1).

## Results

### Global level

**Fig. 1:**
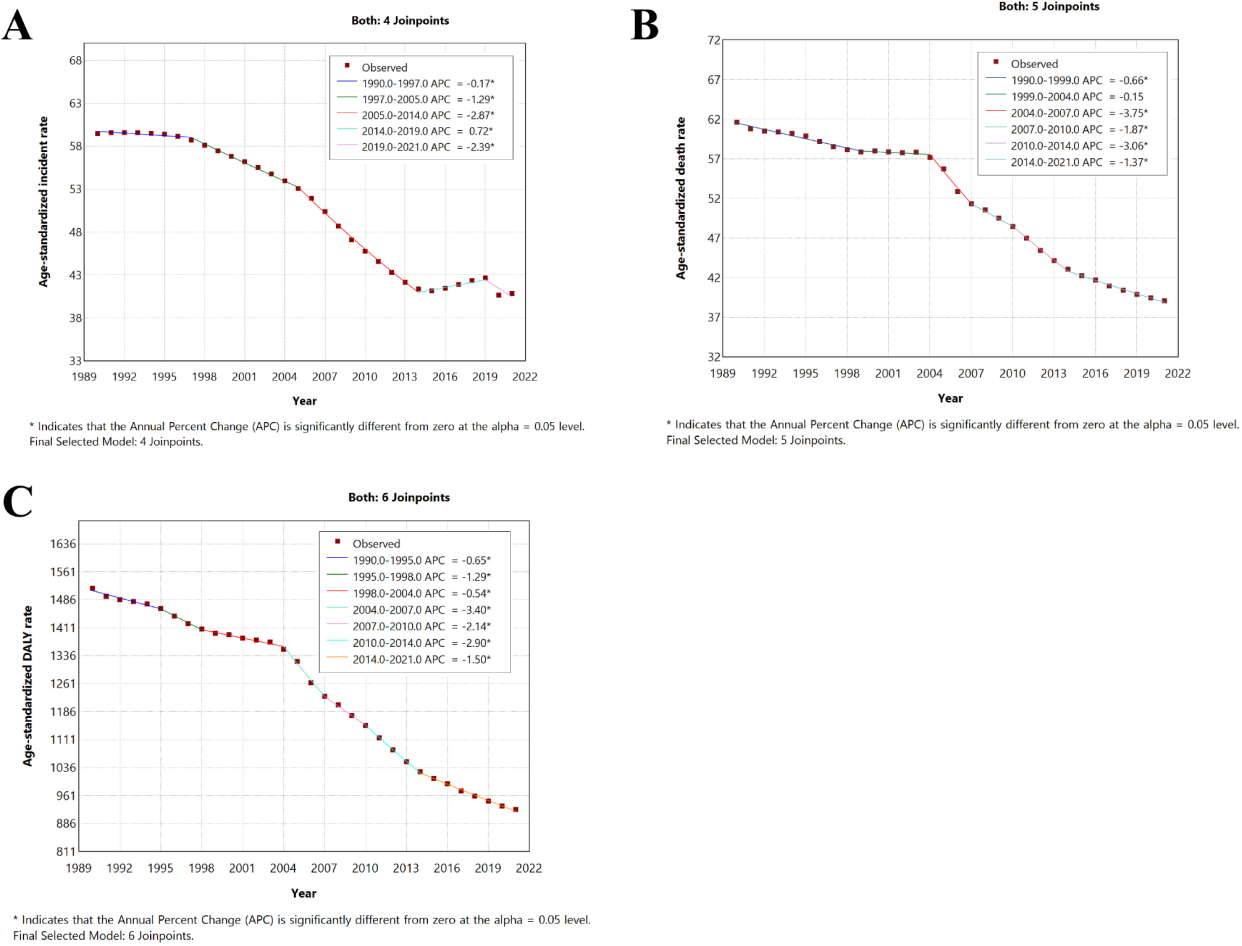
Global trends for age-standardized rates (per 100,000 population) of intracerebral hemorrhage from 1990 to 2021. (**A**) Age-standardized incidence rate; (**B**) Age-standardized death rate; (**C**) Age-standardized DALY rate. *Abbreviations* DALY disability-adjusted life-year

In 2021, 3.444 million new cases of ICH were reported globally, with an age-standardized prevalence rate of 40.8 per 100,000 people, reflecting a 31.4% decrease from 1990. ICH resulted in 3.308 million deaths in 2021, with an age-standardized mortality rate of 39.1 per 100,000 people, marking a 36.6% decrease since 1990. In 2021, the global DALYs attributable to ICH totaled 79.457 million, with an age-standardized DALY rate of 92.4 per 100,000 people, indicating a 39.1% decrease since 1990 (Table 1). Over the past 32 years, the global age-standardized incidence rate (ASIR), death rate, and disability-adjusted life year (DALY) rate of intracerebral hemorrhage have significantly decreased. Specifically, as shown in Fig. 1, the global ASIR markedly declined from 1990 to 2014, increased from 2014 to 2019 (APC = 0.72%, 95% CI: 0.50–0.94%, *p* < 0.05), and then decreased again. Similarly, the age-standardized death rate (ASDR) and DALY rate of intracerebral hemorrhage also significantly decreased, with the most notable declines occurring between 2004 and 2007 (ASDR: APC = -3.75%, 95%CI: -4.52% to -2.97%, *p* < 0.05; DALY rate: APC = -3.40%, 95% CI: -3.97% to -2.82%, *p* < 0.05) (Table 1; Fig. 1).

**Table 1:** The results of estimating the temporal trends in the global burden of intracerebral hemorrhage using the Joinpoint regression model

	Segment	Segment Start	Segment End	APC (95%UI)	P
Incidence	0	1990	1997	-0.17(-0.28 to -0.06)	0.005
	1	1997	2005	-1.29(-1.39 to -1.18)	<0.001
	2	2005	2014	-2.87(-2.95 to -2.79)	<0.001
	3	2014	2019	0.72(0.50 to 0.94)	<0.001
	4	2019	2021	-2.39(-3.13 to -1.64)	<0.001
Death	0	1990	1999	-0.66(-0.75 to -0.56)	<0.001
	1	1999	2004	-0.15(-0.42 to 0.12)	0.247
	2	2004	2007	-3.75(-4.52 to -2.97)	<0.001
	3	2007	2010	-1.87(-2.70 to -1.04)	<0.001
	4	2010	2014	-3.06(-3.54 to -2.57)	<0.001
	5	2014	2021	-1.37(-1.53 to -1.21)	<0.001
DALY	0	1990	1995	-0.65(-0.82 to -0.49)	<0.001
	1	1995	1998	-1.29(-1.92 to -0.66)	<0.001
	2	1998	2004	-0.54(-0.67 to -0.40)	<0.001
	3	2004	2007	-3.40(-3.97 to -2.82)	<0.001
	4	2007	2010	-2.12(-2.73 to -1.55)	<0.001
	5	2010	2014	-2.90(-3.25 to -2.55)	<0.001
	6	2014	2021	-1.50(-1.62 to 1.39)	<0.001

*Abbreviations* APC: annual percentage change; DALY: disability-adjusted life year

### Regional level

In 2021, Central Asia (74.7), Oceania (74.4), and Southeast Asia (69.8) had the highest age-standardized prevalence rates of ICH per 100,000 people, while Australasia (10.8), high-income North America (13.1), and Western Europe (13.2) had the lowest rates. Oceania (110.4), Southeast Asia (80.7), and Central Sub-Saharan Africa (77.1) had the highest age-standardized mortality rates of ICH, while Australasia (6.5), Western Europe (8.1), and high-income North America (9.3) had the lowest. In 2021, the highest age-standardized DALY rates per 100,000 people were reported in Oceania (2582.5), Southeast Asia (1976.8), and Central Sub-Saharan Africa (1772.6), while the lowest rates were seen in Australasia (126.2), Western Europe (161.2), and high-income North America (221.2) (Supplementary Table 1).

From 1990 to 2021, age-standardized incident rates of ICH decreased across all 21 global regions, with the largest declines in Tropical Latin America (-59.9%), high-income Asia Pacific (-55.7%), and the Caribbean (-52.8%). In terms of age-standardized mortality rates, only Sub-Saharan Africa showed an increase of 2.8%. From 1990 to 2021, age-standardized DALY rates for ICH decreased across all regions, with the largest reductions in high-income Asia Pacific (-67.0%), Tropical Latin America (-66.0%), and Southern Latin America (-65.9%) (Supplementary Table 1).

### National level

**Fig. 2:**
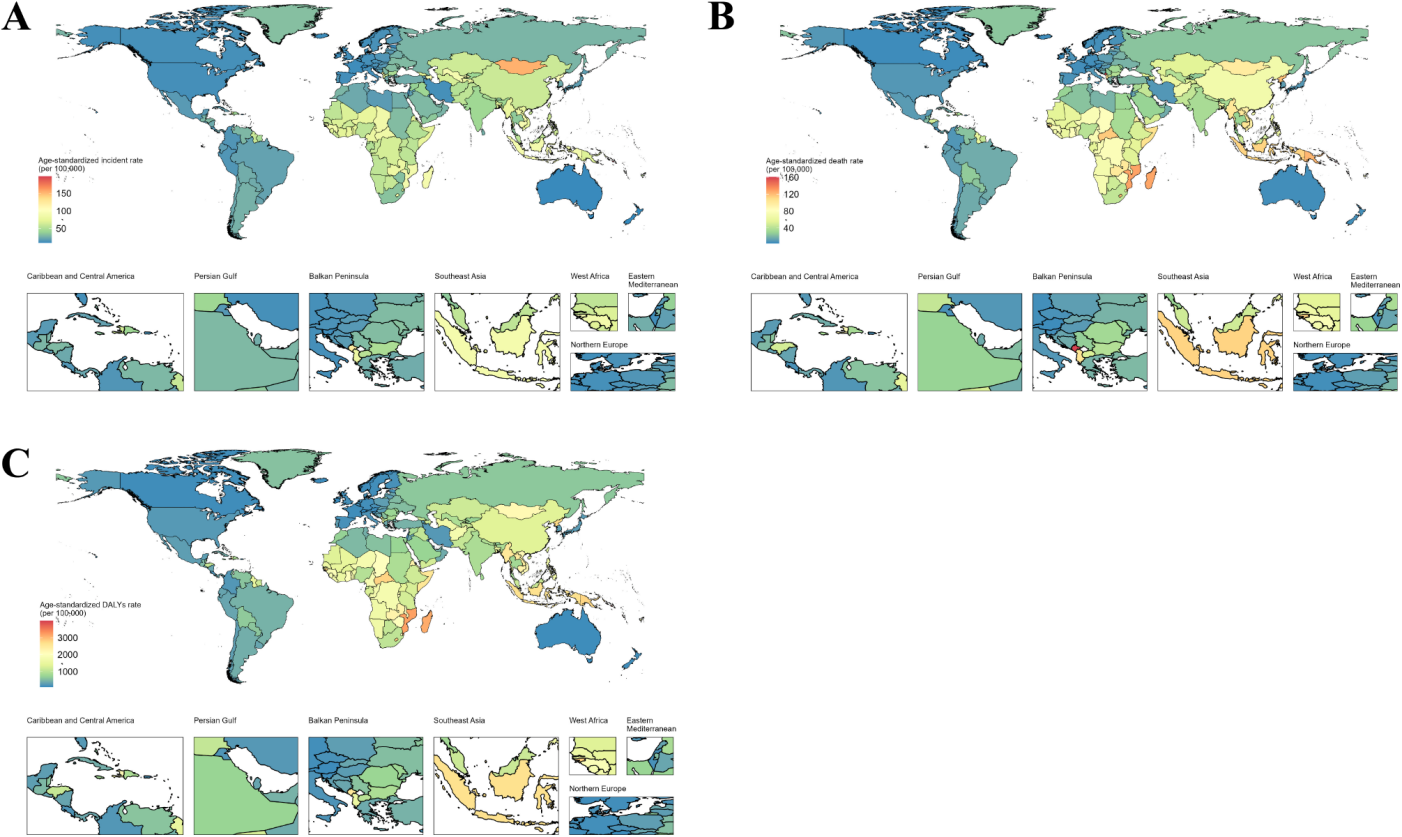
Age-standardised incidence, death and DALY rate of intracerebral hemorrhage per 100 000 population in 2021, by country. (**A**) Age-standardised incident rate; (**B**) Age-standardised death rate; (**C**) Age-standardised DALY rate. Abbreviations: DALY disability-adjusted life-year

In 2021, age-standardized incidence rates of ICH across countries ranged from 9.9 to 198.1 per 100,000 people. The highest rates were observed in the Solomon Islands (198.1), Mongolia (153.3), and Kiribati (140.6), while the lowest rates were in Switzerland (9.9), New Zealand (10.1), and Australia (10.9) (Fig. 2A, Supplementary Table 2). In 2021, age-standardized mortality rates of ICH across countries ranged from 4.3 to 160.7 per 100,000 people. The highest mortality rates were in the Solomon Islands (160.7), Montenegro (159.3), and Nauru (147.2), while the lowest rates were in Switzerland (4.3), Andorra (5.1), and Ireland (5.1) (Fig. 2B and Supplementary Table 3). In 2021, age-standardized DALY rates of ICH across countries ranged from 103.4 to 3998.5 per 100,000 people. The highest rates were in Nauru (3998.5), the Solomon Islands (3737.7), and the Marshall Islands (3450.4), while the lowest rates were in Ireland (103.4), Andorra (108.2), and Iceland (115.8) (Fig. 2C and Supplementary Table 4).

From 1990 to 2021, the largest increases in age-standardized incidence rates of ICH were in Lesotho (32.5%), the Philippines (29.8%), and Uzbekistan (7.2%), while the largest decreases were seen in South Korea (-77.1%), Portugal (-69.6%), and Estonia (-68.7%) (Supplementary Table 2). During the same period, the largest increases in age-standardized mortality rates of ICH were in Cuba (42.7%), Lesotho (35.7%), and Turkmenistan (24.1%), while the largest decreases were in South Korea (-88.2%), Singapore (-80.2%), and Taiwan province of China (-79.2%) (Supplementary Table 3). The largest increases in age-standardized DALY rates of ICH were in Zimbabwe (56.2%), Lesotho (46.3%), and Turkmenistan (22.3%), while the largest decreases were in South Korea (-87.4%), Singapore (-79.3%), and Hungary (-77.7%) (Supplementary Table 4).

### Age and sex patterns

**Fig. 3:**
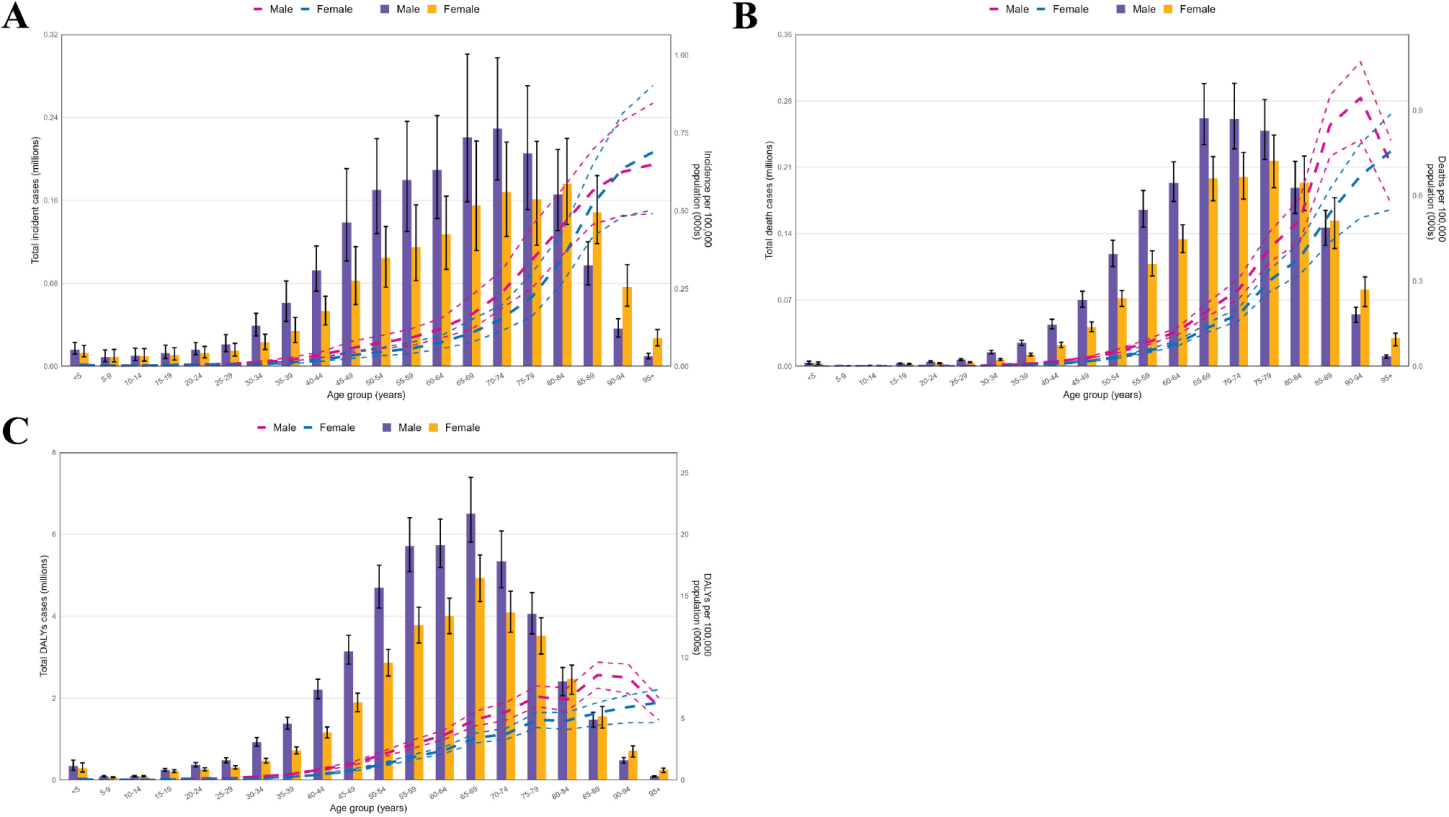
Number of incidence, death and DALYs cases globally and incidence, death and DALYs rate of intracerebral hemorrhage per 100 000 population, by age and sex in 2021. Lines indicate prevalent case with 95% uncertainty intervals for men and women. (**A**)Incidence; (**B**)death; (**C**)DALYs. *Abbreviations* DALY disability-adjusted life-year

In 2021, the global incidence rate of ICH began to significantly increase in the 40–44 age group and peaked in the ≥ 95 age group. The highest number of new cases worldwide in 2021 was in the 70–74 age group, followed by a decline with increasing age. In 2021, the number of new ICH cases among men was higher up to the age of 75–79, but it was more common in women aged 80 and above (Fig. 3A). In 2021, the global mortality rate of ICH reached its highest level in the 90–95 age group, with mortality rates higher in men than in women across all age groups. The highest number of deaths occurred in the 70–74 age group, followed by a decline with increasing age (Fig. 3B). The global DALY rates for ICH in both men and women peaked in the 85–89 age group and then declined with increasing age, with DALY rates higher in men than in women across all age groups (Fig. 3C)

### Association with socio-demographic index

**Fig. 4:**
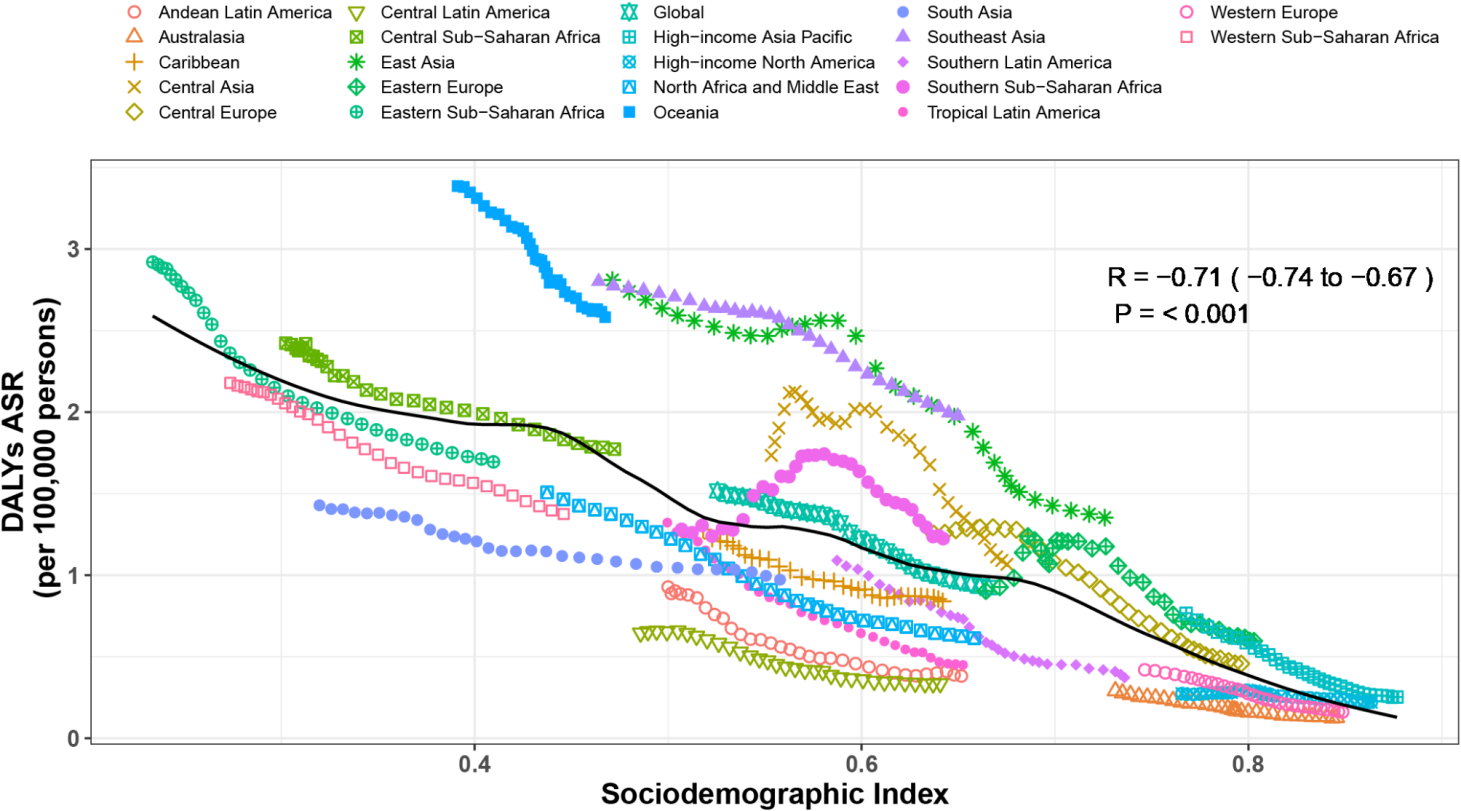
Age-standardised disability adjusted life year (DALY) rates of intracerebral hemorrhage for the 21 Global Burden of Disease regions by sociodemographic index, 1990–2021. Thirty-two points are plotted for each region and show the observed age standardised DALY rates from 1990 to 2021 for that region. Expected values, based on sociodemographic index and disease rates in all locations, are shown as a solid line. Regions above the solid line represent a higher than expected burden

At the regional level, from 1990 to 2021, we observed a significant decrease in the age-standardized DALY rate for intracerebral hemorrhage with increasing SDI (*P* ≤ 0.001). During this period, age-standardized DALY rates in Oceania, East Asia, Southeast Asia, Central Asia, and high-income Asia Pacific were higher than expected. In contrast, the burden in Andean Latin America, Tropical Latin America, the Caribbean, South Asia, Western Sub-Saharan Africa, Australasia, Southern Latin America, and Central Latin America was lower than expected (Fig. 4)

At the national level in 2021, the age-standardized DALY rate for intracerebral hemorrhage decreased gradually with increasing SDI up to approximately 0.35. Beyond an SDI of approximately 0.35, the age-standardized DALY rate for intracerebral hemorrhage decreased rapidly with further increases in SDI (*P* < 0.001). Countries and regions such as the Solomon Islands, Nauru, the Marshall Islands, Vanuatu, and Palau had a significantly higher burden of intracerebral hemorrhage than expected, whereas Niger, Yemen, Cuba, Palestine, and Spain had a significantly lower burden than expected (Supplementary Fig. 1).

### Risk factors

**Fig. 5:**
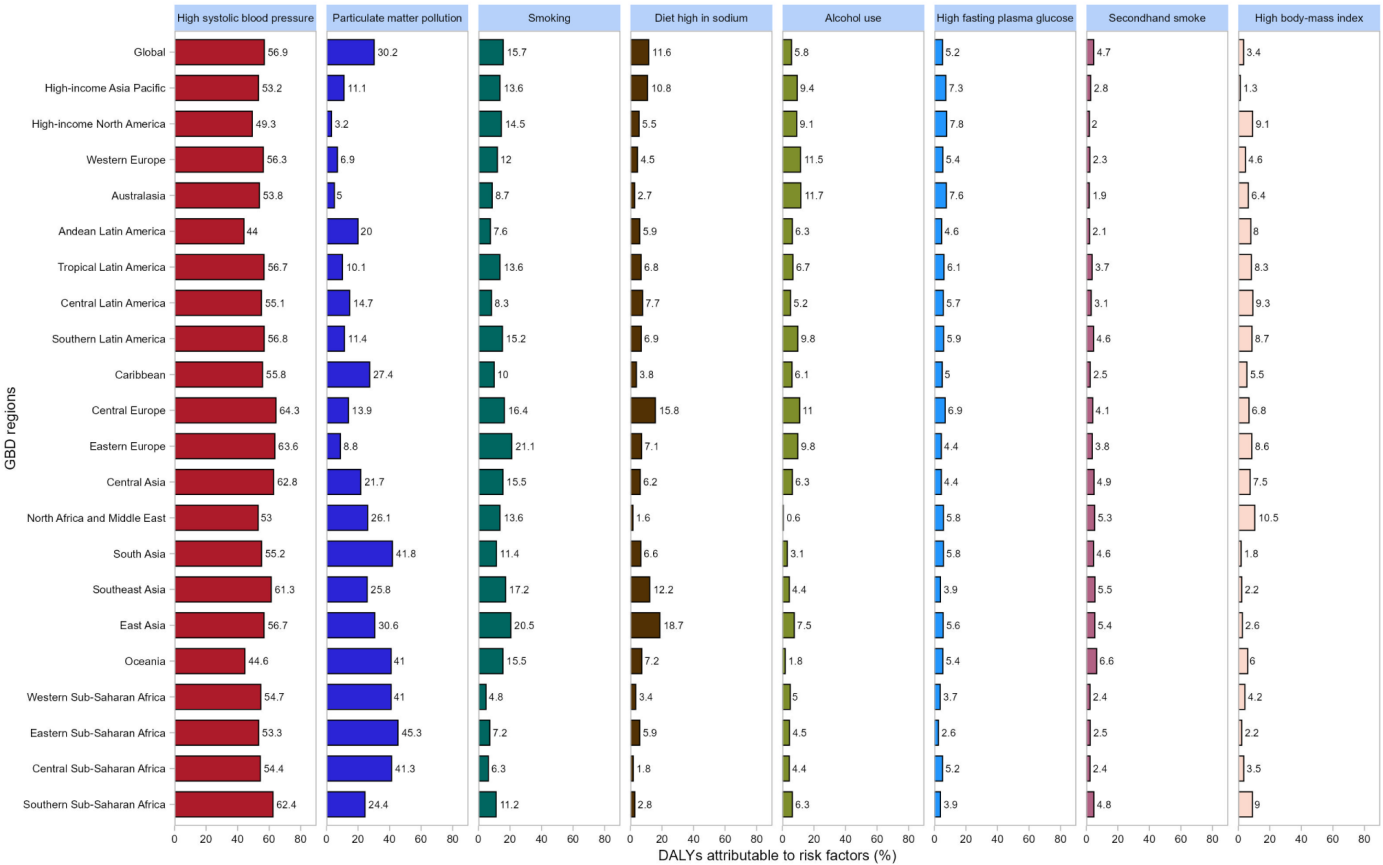
Percentage of disability adjusted life years (DALYs) due to intracerebral hemorrhage attributable to each risk factor for the 21 Global Burden of Disease regions in 2021

The proportion of DALYs attributed to individual risk factors for ICH varies across different GBD regions. Globally, hypertension, particulate matter air pollution, and smoking are the primary contributors to intracerebral hemorrhage-related DALYs. Hypertension is the leading contributor to DALYs, followed by particulate matter air pollution and smoking (Fig. 5).

The charts display the proportion of DALYs due to individual risk factors for ICH across genders. The contribution of risk factors differs between males and females. For example, smoking, sodium intake, and alcohol consumption contribute more to intracerebral hemorrhage-related DALYs in males. However, hypertension and particulate matter air pollution are major risk factors for both sexes. Identifying and managing these key risk factors can lead to more effective prevention strategies to reduce the burden of ICH (Supplementary Figs. 2 and 3).

## Discussion

This study comprehensively examines the global, regional, and national burden of ICH by analyzing data from the GBD database spanning 1990 to 2021. The results indicate that although the global ASIR, ASDR, and age-standardized DALY rate of ICH have declined over the past decades, significant regional and national differences persist. The results of this study are largely consistent with previous research, showing a decline in the global burden of intracerebral hemorrhage. However, by updating the data to 2021, this study provides the latest epidemiological trends and risk factor analysis, filling the data gap from 2019 to 2021. This update is particularly important given that, since the onset of the COVID-19 pandemic in late 2019, healthcare resources have been primarily allocated to controlling and treating the pandemic, making the update of epidemiological data for other diseases crucial. The latest two years of data can help policymakers identify the most recent risk factors and trends, thereby enabling more effective prevention and intervention strategies.

First, the global ASIR for ICH has significantly decreased since 1990, with 3.444 million new cases reported worldwide in 2021 and an age-standardized prevalence rate of 40.8 per 100,000, representing a 31.4% decrease from 1990. This trend is particularly evident in high-income and upper-middle-income regions, which benefit from developed healthcare systems and early intervention measures. However, during the period from 2014 to 2019, the age-standardized incidence rate slightly increased, likely due to the global aging trend, which resulted in an increase in the elderly population and consequently more cases of intracerebral hemorrhage. Additionally, in some regions, enhanced diagnosis and reporting of intracerebral hemorrhage during this period contributed to the rise in incidence rates. In contrast, in low- and middle-income regions such as Sub-Saharan Africa, the decline in ASIR and ASDR has been less pronounced. This is primarily related to the scarcity of medical resources, insufficient public health service coverage, and lower levels of socioeconomic development [16, 17]. Additionally, regarding the global mortality of ICH, in 2021, ICH caused 3.308 million deaths worldwide, accounting for 96% of the total new cases. This high mortality rate is significantly higher than the 85% (2.89 million deaths out of 3.41 million new cases) reported in the previous GBD stroke report. One possible reason for this discrepancy is that different data sources and reporting methods may lead to variations in mortality rates. The GBD 2021 database has a broader data collection scope, including more low- and middle-income countries, where medical resources are relatively scarce and the treatment and rehabilitation conditions for ICH patients are poor, resulting in higher mortality rates. Secondly, during the COVID-19 pandemic, healthcare resources were primarily allocated to combating the pandemic, impacting the diagnosis and treatment of other diseases, which may have also contributed to the increased mortality rate of ICH patients. During the pandemic, many countries’ healthcare systems were under tremendous pressure, and ICH patients might not have received timely and effective treatment, thereby increasing mortality.

At the regional level, in 2021, Central Asia, Oceania, and Southeast Asia had the highest age-standardized incidence rates of ICH at 74.7, 74.4, and 69.8 per 100,000, respectively, while Australasia, high-income North America, and Western Europe had the lowest rates. This reflects disparities in ICH prevention and treatment across regions. High-incidence regions commonly have high prevalence of hypertension, relative scarcity of medical resources, and insufficient health education [18]. For example, Central Asia and Southeast Asia have higher rates of hypertension combined with unhealthy lifestyles, such as high-salt diets and smoking, which further exacerbate the burden of ICH [19].

At the national level in 2021, the Solomon Islands, Mongolia, and Kiribati had the highest age-standardized incidence rates of ICH, while Switzerland, New Zealand, and Australia had the lowest. These differences reflect not only variations in health services and disease prevention but also the impact of socioeconomic development on disease burden [20]. High-incidence countries often face challenges such as scarce medical resources, insufficient health service coverage, and prevalent high-risk behaviors (e.g., smoking and high-salt diets).

Although the global ASDR for ICH generally declined from 1990 to 2021, regions such as Southern, Eastern, and Southeastern Sub-Saharan Africa experienced increases in ASDR and DALY rates. This indicates significant deficiencies in ICH prevention and management in these regions, likely due to limited medical resources, poor accessibility to health services, and suboptimal socioeconomic conditions [21]. Future efforts should focus on fostering regional cooperation to integrate medical advancements from high-income countries into middle- and low-income countries, thereby enhancing their healthcare capabilities [22, 23]. Additionally, the global aging population has contributed to the increased burden of ICH in these regions. Strengthening healthcare infrastructure and public health services, improving hypertension management, reducing smoking rates, and enhancing dietary habits are crucial to reducing the burden of ICH in these regions.

Regarding risk factors, this study confirms that hypertension, particulate matter pollution, and smoking are primary contributors to ICH. As the most important controllable risk factor, effective hypertension management is crucial for ICH prevention. Additionally, smoking and particulate matter pollution significantly increase ICH risk. Compared to the risk factors identified in GBD 2019, high systolic blood pressure remains the most significant risk factor, highlighting the importance of continuous hypertension management. The impact of particulate matter pollution remained significant in 2021, indicating the ongoing necessity of environmental governance. Additionally, the influence of smoking increased in 2021, further underscoring the need for enhanced tobacco control measures. We have also investigated the differences in risk factors across genders, offering valuable references for future prevention strategies tailored to different groups.

The significant association between the SDI and ICH burden was also confirmed in this study. Our research shows that as SDI increases, the age-standardized DALY rate of ICH significantly decreases. Countries and regions with higher SDI tend to have better medical resources and higher health awareness, enabling more effective prevention and treatment, thereby reducing the disease burden. Conversely, countries and regions with lower SDI face greater ICH burdens due to insufficient resources and services. This finding underscores the importance of improving socioeconomic conditions to alleviate the burden of ICH.

In summary, this study provides a comprehensive understanding of the current status and trends of the global ICH burden through a systematic analysis of the GBD database. Although the global ASIR and ASDR for ICH are generally declining, significant regional and national differences still warrant attention. Future public health policies should focus on high-risk regions and populations by implementing comprehensive measures such as improving hypertension management, smoking control, environmental quality, and healthcare service coverage to reduce ICH incidence and mortality and alleviate the global disease burden. Particularly in low- and middle-income countries and regions, increasing medical resource investment, healthcare service coverage, hypertension control rates, reducing smoking rates, and enhancing environmental quality are essential to improving public health. International cooperation and policy support can more effectively address the global challenges of ICH, ultimately leading to a comprehensive improvement in global health levels.

The strengths of this study lie in the use of large-scale global data, covering long-term trend analysis from 1990 to 2021, providing a comprehensive picture of the ICH burden. However, the study also has limitations, such as possible inadequacies in data quality and coverage in some low-income countries, and the assumptions of statistical models may not fully reflect the complexity of real-world situations. Future research should aim to improve data collection and analysis methods to more accurately assess the global and regional ICH burden and develop more effective prevention and control strategies.

## Electronic supplementary material

Below is the link to the electronic supplementary material.

Supplementary Material 1

## Data Availability

Data supporting the findings of this study are available from the Global Burden of Disease (GBD) database. The data can be accessed at https://vizhub.healthdata.org/gbd-results.
