# Supplementary material for: Global, regional, and national burden of intracerebral hemorrhage and its attributable risk factors from 1990 to 2021: results from the 2021 Global Burden of Disease Study
Source: BMC Public Health. 2024 Sep 6;24:2426. doi: 10.1186/s12889-024-19923-7 (PMC11378620; doi:10.1186/s12889-024-19923-7)

Supplementary Table 1: Incidence cases, deaths, and disability adjusted life years (DALYs) for intracerebral hemorrhage in 2021, and percentage change in age-standardised rates (ASRs) per 100000, by Global Burden of Disease region, from 1990 to 2021.

Location	Incidence cases (95% UI)	ASIR (95% UI)	ASIR Change (1990-2021) (95% UI)	Death case (95% UI)	ASDR (95% UI)	ASDR Change (1990-2021) (95% UI)	DALYs cases (95% UI)	ASR DALY (95% UI)	ASR DALY Change (1990-2021) (95% UI)
Global	3444.3 (3053, 3812)	40.8 (36.2,45.2)	-31.4 (-33.7, -28.7)	3308.4 (3021.1, 3594.7)	39.1 (35.6, 42.5)	-36.6 (-42.5, -29.8)	79457.4 (72748.9, 85480.2)	923.6 (844.8, 993.2)	-39.1 (-44.7, -33.1)
High-income Asia Pacific	77.3 (67.8, 85.5)	20 (17.7, 22.5)	-55.7 (-58.3, -52.9)	54.9 (45.4, 60.8)	10.2 (8.8,11)	-67.9 (-70.2, -65.9)	1038.3 (916.7, 1124)	253 (232.8, 271.5)	-67 (-68.9, -64.9)
High-income North America	79.6 (68.3, 90.3)	13.1 (11.3, 14.7)	-26.7 (-30.6, -22.1)	62.8 (56.0, 66.6)	9.3 (8.4,9.8)	-15.3 (-19.3, -12.1)	1343.4 (1250.2, 1412.2)	221.2 (208.2,232.8)	-19.4 (-22.6, -16.6)
Western Europe	124.3 (111.6, 135.1)	13.2 (11.9, 14.5)	-42.7 (-44.9, -40.7)	88.7 (76.2, 95.4)	8.1 (7.1,8.6)	-56.9 (-59.8, -54.7)	1488.4 (1347.1, 1577.9)	161.2 (149.8,170.2)	-61.6 (-63.5, -59.8)
Australasia	5.7 (5.1, 6.2)	10.8 (9.6, 11.9)	-42.2 (-45.4, -39)	3.9 (3.4, 4.3)	6.5 (5.7,7.2)	-52.3 (-56.4, -48.2)	67.8 (61.3, 73.4)	126.6 (115.9,136.7)	-56.5 (-59.8, -53.1)
Andean Latin America	10.8 (9.9, 11.9)	18 (16.3, 19.6)	-49.4 (-51.5, -47.4)	8.8 (7.3, 10.7)	14.9 (12.4,18.1)	-56.8 (-65.4, -46.1)	234.3 (194.0, 284.6)	381.9 (316.4,463.7)	-58.8 (-67, -48.8)
Tropical Latin America	52.7 (45.3, 60.2)	20.8 (17.9, 23.7)	-59.9 (-62.1, -57.4)	43.9 (41, 46)	17.1 (16,17.9)	-64.7 (-66.5, -63.1)	1172.4 (1118.6, 1218.7)	449.7 (428.7,467.4)	-66 (-67.7, -64.5)

Central Latin America	48.3 (43.2, 53.2)	19.6 (17.6, 21.6)	-41.9 (-43.5, -40)	33.5 (29.7, 37.9)	13.7 (12.1, 15.4)	-48.2 (-53.7, -42.2)	853.7 (762.6, 971.2)	335.5 (299.9,381.5)	-48 (-53.8, -41.5)
Southern Latin America	21.6 (19.5, 23.5)	25.8 (23.4, 28.3)	-51.5 (-54, -49.2)	13.2 (12.2, 14.0)	15 (13.9, 15.9)	-64.1 (-66.9, -61.5)	314.1 (296.0, 332.4)	372.1 (350.5, 394)	-65.9 (-68.3, -63.5)
Caribbean	17.6 (16.2, 18.9)	33.3 (30.7, 35.9)	-28 (-29.9, -26.1)	16.7 (14.3, 19.4)	31.2 (26.6, 36.1)	-35.4 (-44.2, -25.1)	443.4 (375.4, 519.2)	839.6 (709.9, 983.2)	-34.4 (-43.9, -23.1)
Central Europe	50.1 (46.0, 53.8)	23.7 (22.0, 25.4)	-52.8 (-54.0, -51.6)	48 (44.2, 51.5)	21 (19.3, 22.5)	-61.7 (-64.5, -58.6)	957.7 (882, 1030)	457.1 (419.7, 492.7)	-63.6 (-66.3, -60.6)
Eastern Europe	92.4 (80.1, 103.8)	28.7 (25.2, 32.4)	-33.7 (-37.0, -30.3)	78.3 (72.1, 84.3)	22.8 (21.0, 24.6)	-38 (-42.5, -33.2)	1930.3 (1777.2, 2078.2)	596.6 (549.1, 642.4)	-34 (-39.3, -29)
Central Asia	55.5 (51.3, 59.6)	74.7 (69.3, 79.9)	-13.4 (-16.0, -10.3)	35 (31.6, 38.8)	47.9 (43.3, 52.9)	-34.6 (-41.4, -27.5)	884.5 (792.9, 985.1)	1066.6 (961.2,1184.2)	-38.5 (-44.8, -31.9)
North Africa and Middle East	121.3 (109.8, 132.3)	25.9 (23.6, 28.1)	-43.3 (-45.1, -41.5)	106.1 (91.1, 121.4)	25.4 (21.6, 28.9)	-56.2 (-62, -49.0)	3051.9 (2633.1, 3493.6)	614.4 (529.3,701.5)	-59.2 (-65.1, -52.3)
South Asia	703.2 (607.0, 804.8)	47.6 (41.0, 54.1)	-29.2 (-32.4, -25.1)	558.3 (456.8, 640.4)	39.4 (32.0, 45.3)	-30.2 (-39.9, -18.8)	15045.6 (12776.9, 17158.1)	972.4 (816.1,1110.7)	-31.9 (-41, -21.2)

Southeast Asia	450.6 (401.0, 497.1)	69.8 (62.4, 76.2)	-25.5 (-27.5, -23.3)	500.8 (439.0, 571.7)	80.7 (70.6, 92.4)	-28.6 (-38.5, -17.7)	13616.7 (12011.1, 15407.5)	1976.8 (1744.3,2238.7)	-29.4 (-38.6, -19.2)
East Asia	1218 (1046.0, 1377.3)	61.1 (53.1, 68.8)	-43.7 (-46.5, -40.5)	1366.6 (1147.3, 1610.9)	68.3 (57.4, 80.2)	-50.5 (-59.9, -40.1)	28529.1 (23808.3, 33766.2)	1353.1 (1138.3,1594)	-51.8 (-60.6, -41.0)
Oceania	5.4 (4.9, 6.0)	74.4 (68.0, 80.5)	-21.6 (-25.4, -17.4)	7.3 (5.8, 8.9)	110.4 (88.8, 133.7)	-23 (-37.7, -3.8)	211.1 (167.0, 258.9)	2582.5 (2058.7,3157.5)	-23.7 (-39.4, -3.7)
Western Sub-Saharan Africa	139 (125.3, 152.6)	65.3 (58.5, 71.5)	-26.9 (-29.6, -24.0)	105.5 (83.7, 126.8)	56.7 (44.4, 67.2)	-36.3 (-45.8, -25.1)	3223.4 (2546.5, 3936.8)	1375.3 (1095,1646.3)	-36.9 (-46.0, -25.4)
Eastern Sub-Saharan Africa	112.9 (102.5, 124.1)	62.2 (56.5, 67.9)	-38.2 (-40.3, -36.0)	111.9 (95.0, 130.1)	71.8 (60.8, 83.4)	-40.1 (-49.0, -30.9)	3231.8 (2741.9, 3779.9)	1694.9 (1441.6,1964.7)	-42 (-51.8, -32.7)
Central Sub-Saharan Africa	37.3 (33.8, 41.2)	63.6 (58.1, 69.4)	-29.4 (-32.6, -25.7)	36.8 (27.7, 47.0)	77.1 (58.6, 99.5)	-22 (-38.5, -1.0)	1064.7 (797.7, 1357.6)	1772.6 (1344.9,2260.3)	-26.8 (-42.6, -6.4)
Southern Sub-Saharan Africa	20.7 (18.2, 23.3)	36.6 (32.1, 40.9)	-29 (-32.2, -25.7)	27.5 (25.4, 29.8)	50.7 (46.8, 55.1)	2.8 (-8.0, 19.3)	754.9 (693.7, 820.8)	1224.2 (1130.5,1326.8)	-4.5 (-14.0, 7.7)

95% UI=95% uncertainty intervals. ASR: Age-standardized rate

Supplementary Table 2: Incidence of Intracerebral Hemorrhage in 1990 and 2021 and the percentage change in the age-standardised rates (ASRs) per 100,000, by location.

location	Number(95%_UI) (1990)	ASR/100000(95%_UI) (1990)	Number(95%_UI) (2021)	ASR/100000(95%_UI) (2021)	Change ASR/100000(1990-2021) (95% UI)
**Global**	2358349 (2052149,2634480)	59.5 (51.4,66.6)	3444338 (3053009,3812043)	40.8 (36.2,45.2)	-31.4 (-33.7, -28.7)
**Andean Latin America**	8396 (7653,9186)	35.5 (32.2,38.8)	10818 (9858,11861)	18 (16.3,19.6)	-49.4 (-51.5, -47.4)
**Bolivia (Plurinational State of)**	1662 (1482,1846)	47 (42.1,51.7)	2234 (2001,2465)	25.3 (22.6,27.7)	-46.2 (-49.1, -43.0)
**Ecuador**	1990 (1808,2195)	32.2 (29.2,35.3)	3087 (2791,3384)	18.8 (16.9,20.6)	-41.7 (-44.9, -38.5)
**Peru**	4744 (4285,5235)	34 (30.5,37.4)	5497 (4956,6081)	15.8 (14.2,17.5)	-53.5 (-56.2, -50.5)
**Australasia**	4256 (3909,4576)	18.6 (17.1,20.1)	5698 (5118,6243)	10.8 (9.6,11.9)	-42.2 (-45.4, -39)
**Australia**	3562 (3288,3821)	18.7 (17.3,20.1)	4867 (4375,5355)	10.9 (9.7,12.1)	-41.8 (-46, -38.1)
**New Zealand**	694 (574,813)	18.1 (15,21)	831 (720,950)	10.1 (8.8,11.5)	-44 (-48.3, -38.6)
**Caribbean**	12489 (11536,13403)	46.2 (42.7,49.5)	17584 (16192,18935)	33.3 (30.7,35.9)	-28 (-29.9, -26.1)
**Antigua and Barbuda**	28 (26,31)	52 (47.8,56.4)	31 (28,34)	31.3 (28.5,33.8)	-39.9 (-43.1, -36.1)
**Bahamas**	75 (69,81)	44.6 (41,48.3)	124 (113,135)	30.9 (28.4,33.6)	-30.7 (-34.1, -26.7)
**Barbados**	106 (97,116)	37.6	116	24.6	-34.5

		(34.3,40.7)	(105,129)	(22.4,27.2)	(-38, -30.5)
**Belize**	39 (36,43)	37.6 (34.4,40.8)	88 (80,97)	28.1 (25.6,30.6)	-25.3 (-29.2, -21.0)
**Bermuda**	17 (15,19)	28.2 (25.2,31.2)	17 (15,19)	14.3 (12.7,16.1)	-49.2 (-53.0, -45.6)
**Cuba**	3654 (3338,3979)	35.7 (32.7,38.8)	3753 (3397,4152)	20.7 (18.8,22.9)	-42.1 (-45.6, -38.4)
**Dominica**	22 (20,24)	36.8 (33.1,39.9)	21 (19,23)	26.1 (23.6,28.7)	-29.0 (-32.9, -25.0)
**Dominican Republic**	1742 (1591,1903)	41.9 (38.4,45.8)	4416 (4033,4793)	43.2 (39.5,46.9)	3.1 (-2.0, 8.0)
**Grenada**	41 (38,45)	57.1 (52.9,61.7)	36 (33,39)	34.1 (31.4,36.7)	-40.2 (-43.4, -36.6)
**Guyana**	505 (468,538)	121.2 (112,129.9)	390 (359,420)	60 (55.4,64.2)	-50.5 (-53.1, -48.3)
**Haiti**	3441 (3107,3730)	99.8 (90.7,108.2)	5047 (4600,5478)	65.4 (59.8,71)	-34.4 (-37.5, -31.4)
**Jamaica**	852 (775,924)	46.8 (42.5,51)	1120 (1022,1221)	36.2 (33.1,39.5)	-22.7 (-26.9, -18.1)
**Puerto Rico**	837 (748,927)	23.7 (21.2,26.2)	894 (792,990)	13.6 (12.1,15.2)	-42.4 (-45.4, -38.5)
**Saint Kitts and Nevis**	34 (31,37)	93.7 (86.6,100.6)	29 (26,32)	43.8 (40.5,47.6)	-53.2 (-55.8, -50.4)
**Saint Lucia**	54 (49,59)	60.9 (56,65.8)	73 (67,79)	31.7 (29.1,34.4)	-48.0 (-50.9, -44.7)
**Saint Vincent and the Grenadines**	30 (28,33)	41.1 (37.6,44.6)	49 (45,53)	35.7 (32.8,38.7)	-13.3 (-18.3, -7.4)
**Suriname**	163 (149,175)	60.5 (55.4,65)	302 (277,327)	48.4 (44.4,52.4)	-20.0 (-23.7,

					-15.5)
**Trinidad and Tobago**	397 (364,435)	46.3 (42.6,50.4)	443 (399,490)	24.5 (22.1,27)	-47.2 (-50.4, -43.5)
**United States Virgin Islands**	26 (23,29)	30.4 (26.9,33.5)	40 (36,44)	24.4 (22,26.8)	-19.8 (-24.7, -14.6)
**Central Asia**	39621 (36706,42561)	86.3 (80.1,92.5)	55548 (51306,59650)	74.7 (69.3,79.9)	-13.4 (-16, -10.3)
**Armenia**	1137 (1024,1255)	44.6 (40.3,49.2)	782 (702,864)	19.1 (17.2,21.1)	-57.1 (-59.8, -54.3)
**Azerbaijan**	4160 (3831,4521)	87.3 (80.1,94.7)	7623 (6948,8239)	92.6 (84.9, 100.1)	6.1 (0.1, 13.0)
**Georgia**	7161 (6624,7734)	122.1 (113.3,131.5)	4232 (3857,4600)	71.2 (65.3,77)	-41.7 (-44.7, -38.3)
**Kazakhstan**	9268 (8480,10032)	75.9 (69.6,81.9)	9921 (9132,10790)	62.4 (57.2,67.8)	-17.9 (-22.1, -12.2)
**Kyrgyzstan**	2610 (2406,2839)	86.6 (80.1,94)	2156 (1978,2334)	42.7 (39.3,46.4)	-50.6 (-53.6, -47.3)
**Mongolia**	1714 (1570,1847)	162.6 (149.3,174.7)	3384 (3156,3621)	153.3 (142.6,164.8)	-5.7 (-11.3, -0.2)
**Tajikistan**	2217 (2014,2399)	79.8 (72.3,86.5)	4012 (3648,4383)	79.9 (73.2,87.2)	0.1 (5.6,6.8)
**Turkmenistan**	1440 (1318,1565)	73.2 (67.2,79.4)	2775 (2551,2995)	66.1 (60.7,71.6)	-9.8 (-14.3, -4.9)
**Uzbekistan**	9914 (9131,10703)	85.2 (78.6,92.2)	20663 (18954,22275)	91.4 (84,99)	7.2 (0.9, 14.3)
**Central Europe**	70009 (64093,75291)	50.3 (46.3,53.9)	50101 (45976,53823)	23.7 (22,25.4)	-52.8 (-54, -51.6)
**Albania**	1787 (1659,1904)	98.2 (90.9,104.2)	3294 (3034,3554)	81.8 (75.7,87.8)	-16.8 (-21.3, -12.5)
**Bosnia and Herzegovina**	1019 (907,1113)	27.1 (24.3,29.9)	1055 (950,1163)	18.3 (16.5,20.2)	-32.4 (-36.8, -27.1)

**Bulgaria**	11098 (10307,11979)	103.1 (96.4,109.6)	6520 (6090,6940)	48.7 (45.6,51.6)	-52.8 (-54.9, -50.8)
**Croatia**	2504 (2324,2689)	43.4 (40.4,46.5)	1391 (1281,1507)	16.4 (15.1,17.7)	-62.2 (-64.4, -59.8)
**Czechia**	4062 (3643,4457)	31.3 (28.4,34.3)	2832 (2494,3196)	14.4 (12.8,16.2)	-54.1 (-58.1, -49.7)
**Hungary**	6565 (6085,7098)	48.9 (45.4,52.7)	3050 (2731,3369)	17.1 (15.3,19)	-65.1 (-67.5, -62.7)
**Montenegro**	802 (743,860)	138 (128.1,148.1)	1058 (974,1138)	122.1 (113.4,130.6)	-11.5 (-15.7, -6.5)
**North Macedonia**	1280 (1174,1369)	72.2 (66.8,77.5)	1476 (1351,1592)	54.1 (49.8,57.7)	-25.1 (-28.7, -21.3)
**Poland**	14587 (12196,16773)	35.7 (30.1,41)	10413 (8928,11848)	15.6 (13.5,17.6)	-56.2 (-58.8, -53.6)
**Romania**	16218 (14950,17496)	62.1 (57.7,66.8)	11302 (10511,12134)	31.5 (29.3,33.7)	-49.3 (-51.8, -46.8)
**Serbia**	6136 (5582,6657)	61.2 (56.4,66)	4917 (4522,5296)	31 (28.6,33.3)	-49.4 (-52.5, -46.3)
**Slovakia**	2142 (1939,2328)	37.1 (33.7,40.2)	1536 (1394,1684)	17.6 (15.9,19.2)	-52.7 (-55.2, -49.7)
**Slovenia**	690 (615,758)	29.5 (26.4,32.5)	528 (475,582)	12.2 (11.1,13.4)	-58.7 (-61.2, -56)
**Central Latin America**	31601 (28194,35407)	33.8 (30.1,37)	48325 (43161,53249)	19.6 (17.6,21.6)	-41.9 (-43.5, -40.0)
**Colombia**	7079 (6459,7828)	36.8 (33.4,40.4)	8568 (7662,9538)	15.6 (13.9,17.4)	-57.6 (-60.4, -54.2)
**Costa Rica**	526 (473,596)	26.8 (24.1,29.7)	855 (749,960)	16 (14.1,18.1)	-40.2 (-43.9, -36.6)
**El Salvador**	1352 (1236,1484)	39.5 (35.9,42.9)	1246 (1117,1387)	19 (17.1,21)	-51.8 (-54.8,

					-48.6)
**Guatemala**	1768 (1634,1928)	41.1 (38.2,44.2)	3272 (3020,3568)	28.8 (26.7,31.4)	-29.8 (-34.3, -25.3)
**Honduras**	776 (694,857)	33.5 (30,36.9)	1807 (1606,1997)	30.5 (27,33.6)	-8.8 (-13.6, -4.0)
**Mexico**	13976 (11884,16181)	29.1 (24.9,33.4)	21743 (18800,24735)	17.7 (15.4,20.1)	-39.1 (-41.9, -35.5)
**Nicaragua**	728 (653,810)	42.7 (38.2,47.1)	1164 (1042,1294)	23.7 (21.2,26.4)	-44.5 (-47.7, -40.7)
**Panama**	613 (553,673)	38.8 (34.7,42.6)	1003 (898,1103)	22.5 (20.2,24.8)	-42.0 (-45.6, -38.2)
**Venezuela (Bolivarian Republic of)**	4782 (4340,5279)	44.5 (40.1,48.7)	8667 (7808,9554)	29.8 (26.8,32.7)	-33.1 (-36.6, -29.0)
**Central Sub-Saharan Africa**	21106 (18699,23579)	90 (80.5,99.1)	37323 (33809,41188)	63.6 (58.1,69.4)	-29.4 (-32.6, -25.7)
**Angola**	4033 (3586,4496)	95.9 (85.6,105.5)	7307 (6567,8140)	56.3 (50.8,62.3)	-41.3 (-44.9, -37.6)
**Central African Republic**	1326 (1179,1480)	109.9 (98.2,120.2)	2057 (1853,2261)	84.7 (77.3,92)	-22.9 (-26.9, -18.2)
**Congo**	1166 (1040,1302)	104.1 (93.4,114.6)	1710 (1534,1895)	60.3 (54.3,66.1)	-42.1 (-45.1, -38.5)
**Democratic Republic of the Congo**	13892 (12233,15570)	86.3 (76.7,95.4)	25441 (23000,28131)	65.5 (59.5,71.5)	-24.1 (-28.2, -19.0)
**Equatorial Guinea**	203 (183,227)	101.4 (91.3,112)	258 (228,292)	47.1 (41.5,52.4)	-53.6 (-56.8, -49.7)
**Gabon**	486 (432,540)	83.9 (74.8,92.8)	549 (487,617)	51.3 (45.6,56.9)	-38.9 (-42.4, -35.2)
**East Asia**	804381 (673103,927435)	108.6 (91.6,124.1)	1218007 (1046031,1377277)	61.1 (53.1,68.8)	-43.7 (-46.5, -40.5)
**China**	774012	108.9	1173288	61.2 (53,69.1)	-43.9

	(644709,896197)	(91.7,124.9)	(1003993,1330455)		(-46.7, -40.5)
**Democratic People's Republic of Korea**	18046 (16340,19579)	123.1 (111.6,133.9)	30962 (28575,33440)	98.8 (91.5,106.4)	-19.7 (-23.8, -14.7)
**Taiwan (Province of China)**	12324 (11152,13387)	84.6 (75.8,92)	13756 (12119,15397)	35.1 (31.1,39.5)	-58.5 (-61.4, -55.2)
**Belarus**	4733 (4269,5219)	38.7 (35,42.6)	3899 (3525,4278)	26.8 (24.3,29.3)	-30.7 (-34.6, -26.2)
**Estonia**	692 (630,750)	35.7 (32.5,38.5)	276 (242,308)	11.2 (9.8,12.5)	-68.7 (-71.8, -65.3)
**Latvia**	1722 (1589,1866)	49.9 (46.1,53.9)	726 (661,791)	19.4 (17.7,21.2)	-61.1 (-63.5, -58.6)
**Lithuania**	1115 (1018,1208)	25.7 (23.5,27.9)	919 (824,1003)	17.6 (15.8,19.2)	-31.7 (-37.0, -26.7)
**Republic of Moldova**	3499 (3239,3795)	82.5 (76.9,89)	1906 (1743,2086)	33.5 (30.7,36.6)	-59.4 (-61.8, -56.8)
**Russian Federation**	74551 (61574,86196)	44 (36.5,50.5)	63552 (54799,71646)	28.7 (25,32.4)	-34.8 (-38.2, -30.9)
**Ukraine**	27908 (22976,32442)	41.7 (34.9,48.2)	21148 (17998,24397)	31.1 (26.7,35.8)	-25.4 (-30.7, -20.0)
**Eastern Sub-Saharan Africa**	81517 (72264,90930)	100.7 (89.6,111.6)	112870 (102527,124053)	62.2 (56.5,67.9)	-38.2 (-40.3, -36.0)
**Burundi**	3282 (2972,3597)	128.3 (116.1,140.4)	3610 (3278,3947)	66.6 (60.3,73)	-48.1 (-50.9, -45.1)
**Comoros**	191 (170,213)	90.1 (81,99.9)	257 (232,285)	52.1 (47.3,57.1)	-42.2 (-45.7, -38.7)
**Djibouti**	131 (115,146)	82 (73.8,90.9)	367 (323,408)	53.8 (48.1,59.5)	-34.4 (-38.5, -30.3)
**Eritrea**	1550 (1374,1720)	125.4 (112.3,137.3)	2199 (1979,2429)	70.3 (64.5,76.6)	-43.9 (-47.2,

					-40.8)
**Ethiopia**	25572 (21613,29573)	121.5 (102.6,140.6)	23136 (20179,26013)	51.9 (45.1,58.7)	-57.3 (-59.9, -54.2)
**Kenya**	6300 (5377,7243)	70.2 (58.7,80.9)	12822 (11113, 14655)	55 (47.6, 62.3)	-21.6 (-24.9, -17.1)
**Madagascar**	7186 (6599,7759)	125.8 (115.5,136.3)	11932 (10869,13043)	91 (83.3, 99.4)	-27.6 (-30.8, -24.1)
**Malawi**	3271 (2942,3617)	79.7 (71.6,88.2)	4425 (4033,4809)	54.8 (50,59.7)	-31.2 (-34.8, -26.7)
**Mozambique**	6095 (5445,6681)	97.6 (87.3,107.3)	11381 (10389,12383)	89.2 (82,96.5)	-8.6 (-13.6, -2.7)
**Rwanda**	4713 (4238,5162)	153 (137.7,166.4)	4097 (3676,4547)	65.6 (59.1,72)	-57.1 (-59.6, -54.4)
**Somalia**	3248 (2886,3610)	112.1 (100.4,123.1)	5562 (4999,6080)	76 (68.7,82.2)	-32.2 (-35.6, -28.3)
**South Sudan**	2275 (2034,2522)	84.8 (75.8,93.1)	2284 (2047,2548)	57.2 (51.6,62.9)	-32.6 (-36.3, -28.6)
**Uganda**	5569 (5024,6184)	81.5 (73.5,89.7)	9466 (8560,10393)	58.4 (52.8,63.4)	-28.4 (-32.1, -24.3)
**United Republic of Tanzania**	9064 (8236,9956)	78.7 (71.1,86.9)	15215 (13875,16728)	55.6 (50.4,60.7)	-29.4 (-33.3, -25.1)
**Zambia**	3013 (2711,3279)	98.9 (89.2,107.5)	6019 (5534,6545)	81.3 (74.6,88.2)	-17.8 (-22.6, -12.5)
**High-income Asia Pacific**	88022 (75966,99117)	45.2 (39.2,50.7)	77253 (67778,85468)	20 (17.7,22.5)	-55.7 (-58.3, -52.9)
**Brunei Darussalam**	90 (80,102)	71.3 (63,79.9)	124 (110,139)	34.5 (30.7,38.2)	-51.6 (-55.1, -47.8)
**Japan**	54311 (44020,64051)	33.8 (27.7,39.6)	55281 (48068,62043)	19.1 (16.7,21.6)	-43.4 (-47.8, -37.9)
**Republic of**	32437	106 (95.1,117)	20452	24.2	-77.1

**Korea**	(28864,36142)		(17866,22876)	(21.3,27.1)	(-78.9, -75.2)
**Singapore**	1184 (1058,1324)	49 (43.9,54.7)	1396 (1216,1572)	17.2 (15.1,19.2)	-64.9 (-67.8, -62.0)
**High-income North America**	60962 (50848,70525)	17.8 (14.9,20.6)	79595 (68334,90253)	13.1 (11.3,14.7)	-26.7 (-30.6, -22.1)
**Canada**	5728 (5044,6393)	18.2 (16.1,20.2)	8894 (7654,9868)	13.3 (11.4,15)	-27.1 (-33.5, -19.6)
**Greenland**	18 (17,20)	55 (49.4,60)	20 (18,22)	32.7 (29.7,35.7)	-40.5 (-43.9, -36.6)
**United States of America**	55214 (45722,64380)	17.8 (14.7,20.7)	70680 (60482,80921)	13 (11.2,14.8)	-26.7 (-30.8, -21.8)
**North Africa and Middle East**	85082 (76375,93986)	45.7 (41.1,49.9)	121272 (109767,132332)	25.9 (23.6,28.1)	-43.3 (-45.1, -41.5)
**Afghanistan**	6091 (5469,6712)	91 (81.7,99.3)	6824 (6177,7494)	51.8 (47.3,56.2)	-43.1 (-45.7, -40.1)
**Algeria**	6495 (5796,7199)	50.1 (44.8,54.9)	8459 (7545,9362)	23.9 (21.3,26.2)	-52.4 (-54.9, -49.6)
**Bahrain**	86 (76,96)	36.7 (32.9,40.3)	172 (151,196)	18 (16.2,20)	-50.9 (-53.7, -47.7)
**Egypt**	10959 (9595,12318)	36.3 (31.3,40.7)	16886 (15018,18785)	25.8 (23.1,28.2)	-28.9 (-33.8, -23.2)
**Iran (Islamic Republic of)**	6843 (5786,8082)	22.5 (19.2,25.8)	10019 (8702,11432)	13.3 (11.5,15)	-41.2 (-44.2, -37.9)
**Iraq**	5875 (5340,6377)	59.4 (53.8,64.8)	11445 (10431,12444)	43.4 (39.8,46.9)	-27.0 (-31.1, -22.2)
**Jordan**	621 (552,698)	39.2 (35,43.4)	1511 (1354,1705)	19.7 (17.8,21.6)	-49.9 (-53.2, -46.5)
**Kuwait**	168 (146,194)	18 (16.2,20)	587 (525,654)	16.9 (15.1,18.8)	-6.1 (12.5,

					0.6)
**Lebanon**	795 (708,886)	36.7 (32.5,40.6)	1117 (991,1245)	18.4 (16.3,20.6)	-49.8 (-52.6, -47)
**Libya**	666 (592, 746)	26.1 (23.4, 28.8)	1031 (925, 1143)	18.2 (16.2, 20.1)	-30.5 (-34.2, -26.7)
**Morocco**	6940 (6240,7659)	44.7 (40.1,48.9)	9578 (8581,10593)	29 (26.1,32)	-35.1 (-38.4, -31.1)
**Oman**	417 (374,462)	47.7 (42.7,52.6)	622 (557,688)	28.1 (25.4,31.1)	-41.1 (-44.8, -37.0)
**Palestine**	446 (403,492)	50.2 (45.3,54.8)	683 (621,749)	29.6 (27.1,31.9)	-41.0 (-44.6, -37.0)
**Qatar**	97 (87,108)	46.9 (42.1,51.5)	340 (291,391)	25.7 (23.2,28.1)	-45.3 (-48.7, -41.9)
**Saudi Arabia**	3308 (2975,3641)	49.8 (44.5,54.3)	7381 (6725,8117)	30.1 (27.6,32.5)	-39.5 (-43.3, -35.9)
**Sudan**	6489 (5851,7155)	62 (55.7,68)	7239 (6482,7973)	31.5 (28.4,34.5)	-49.2 (-51.8, -46.5)
**Syrian Arab Republic**	4327 (3961,4699)	65.2 (59.6,70)	4653 (4240,5064)	39.8 (36.4,42.8)	-39 (-42.0, -36.1)
**Tunisia**	1748 (1555,1956)	35.1 (31.2,38.7)	2696 (2377,3014)	21.7 (19.2,24.1)	-38 (-41.4, -34.0)
**Turkey**	18699 (16824,2063 0)	50.2 (45.1,55.2)	22173 (19642,2461 2)	25.1 (22.2,27.8)	-50.0 (-53.3, -46.6)
**United Arab Emirates**	360 (315,409)	50.6 (45.3,55.5)	1826 (1559,2116)	29 (25.9,32.1)	-42.7 (-46.2, -38.7)
**Yemen**	3605 (3225,3991)	64.3 (57.4,70.4)	5915 (5357,6478)	36.4 (33.2,39.5)	-43.5 (-46.4, -40.2)
**Oceania**	2657 (2379,2944)	94.9 (85.7,103.6)	5443 (4926,5964)	74.4 (68,80.5)	-21.6 (-25.4, -17.4)
**American**	21 (19,23)	90.7	29 (26,32)	65.4	-27.9

**Samoa**		(81.7,98.9)		(59.6,71.6)	(-32.0, -23.4)
**Cook Islands**	9 (8,10)	73.5 (65.7,82)	11 (10,12)	45.9 (40.9,50.9)	-37.5 (-41.7, -33.4)
**Fiji**	323 (291,354)	85.4 (76.9,93.1)	383 (348,424)	54 (49.3,59)	-36.7 (-40.5, -32.6)
**Guam**	41 (37,46)	54.6 (49.1,60.7)	82 (74,90)	40.1 (36.1,44)	-26.6 (-31.4, -21.7)
**Kiribati**	82 (74,88)	189.5 (173.2,203.9)	115 (106,123)	140.6 (130.3,150)	-25.8 (-29.3, -22.5)
**Marshall Islands**	25 (23,27)	144.5 (131.1,156.3)	43 (39,47)	121.5 (112.4,130.9)	-15.9 (-19.9, -11.4)
**Micronesia (Federated States of)**	76 (69,83)	152.7 (138,165.7)	88 (79,95)	121.2 (111.2,131.1)	-20.6 (-24.6, -16.6)
**Nauru**	7 (6,8)	131.9 (118.5,144.5)	7 (6,7)	88 (80.1,95.1)	-33.3 (-37.0, -29.7)
**Niue**	2 (2,2)	100 (89.4,109.5)	1 (1,2)	68.8 (62.5,75)	-31.2 (-34.9, -26.8)
**Northern Mariana Islands**	17 (15,20)	82.6 (73.5,91.1)	29 (25,32)	60.6 (54.5,66.5)	-26.6 (-31.7, -21.2)
**Palau**	11 (10,13)	114.4 (102.6,125)	18 (16,20)	85.4 (78.2,93.7)	-25.3 (-29.4, -20.7)
**Papua New Guinea**	1355 (1195,1526)	85.5 (76.1,94.6)	3310 (2954,3647)	69.7 (63,76.3)	-18.5 (-24.0, -12.5)
**Samoa**	94 (85,103)	110 (99.3,120.3)	121 (110,132)	82.1 (74.7,89.6)	-25.4 (-30.3, -20.3)
**Solomon Islands**	300 (269,328)	219.4 (199,238.1)	714 (664,763)	198.1 (184.5,211.6)	-9.7 (-14.2, -4.4)
**Tokelau**	1 (1,1)	101.5 (90.2,111)	1 (1,1)	58.8 (52.6,64.5)	-42.0 (-45.8, -38.0)

**Tonga**	32 (29,36)	60.1 (52.9,66.4)	38 (34,42)	47 (42,52.1)	-21.9 (-26.8, -16.4)
**Tuvalu**	9 (8,9)	134.7 (122.1,145.7)	9 (8,10)	91.2 (84,98.8)	-32.3 (-35.8, -28.6)
**Vanuatu**	82 (72,91)	123.8 (111.2,135.2)	201 (185,218)	109.2 (100.4,118.1)	-11.8 (-16.5, -6.4)
**South Asia**	387268 (328887,446388)	67.2 (56.5,77.2)	703200 (607008,804764)	47.6 (41,54.1)	-29.2 (-32.4, -25.1)
**Bangladesh**	48511 (43817,53208)	99.3 (88.9,109.5)	94996 (85170,103929)	71 (63.9,77.6)	-28.5 (-32.7, -23.2)
**Bhutan**	150 (132,172)	58.8 (51.8,66.3)	224 (197,250)	36.9 (32.4,41.3)	-37.2 (-41.8, -32.3)
**India**	286934 (238809,334799)	62 (51.4,72.3)	523413 (446706,604615)	43.9 (37.4,50.5)	-29.3 (-32.9, -24.6)
**Nepal**	6276 (5453,7077)	65.4 (57.4,73)	10731 (9411,12148)	47.6 (42.2,53.7)	-27.2 (-32.3, -21.5)
**Pakistan**	45396 (37748,52577)	78.2 (64.5,90.3)	73838 (62787,84518)	55.9 (47.7,63.9)	-28.6 (-32.3, -24)
**Southeast Asia**	244779 (215410,272158)	93.7 (82.9,103.7)	450578 (400978,497072)	69.8 (62.4,76.2)	-25.5 (-27.5, -23.3)
**Cambodia**	5209 (4731,5654)	117.6 (107.1,127.3)	10875 (10011,11789)	92.4 (85.6,99.7)	-21.4 (-25.5, -17.7)
**Indonesia**	112617 (95481,129820)	108.4 (90.2,124.1)	207346 (176874,238266)	89.8 (77.7,101.1)	-17.2 (-21.1, -12.1)
**Lao People's Democratic Republic**	2633 (2398,2868)	124.7 (113.8,135.2)	4141 (3786,4470)	87.1 (80.3,93.9)	-30.2 (-34.1, -25.9)
**Malaysia**	9462 (8566,10339)	96.3 (86.6,105.4)	15319 (13747,16799)	56.5 (50.6,61.5)	-41.4 (-45.1, -37.4)
**Maldives**	103 (93,113)	106.5 (96.3,115.3)	162 (146,179)	44.9 (40.3,49.2)	-57.8 (-60.6,

					-54.7)
**Mauritius**	560 (512,612)	73.3 (67.2,79.5)	575 (515,644)	34 (30.6,37.7)	-53.7 (-56.3, -50.4)
**Myanmar**	28923 (26115,31675)	124.6 (112.9,136.1)	38112 (35041,41461)	79.1 (72.8,85.3)	-36.5 (-39.9, -32.4)
**Philippines**	14628 (12302,16908)	49.3 (41,57)	54563 (47210,62616)	63.9 (55.5,72.2)	29.8 (22.9, 39.1)
**Seychelles**	36 (32,39)	62.9 (56.3,68.5)	46 (41,50)	40.3 (36.2,43.9)	-36 (-39.9, -31.6)
**Sri Lanka**	4842 (4300,5424)	45.1 (39.8,50.3)	6495 (5720,7211)	26.5 (23.5,29.1)	-41.3 (-45.2, -37.4)
**Thailand**	24474 (21890,26962)	68.4 (61.2,75.0)	36765 (32600,40278)	36.8 (32.7,40.2)	-46.2 (-49.7, -41.9)
**Timor-Leste**	239 (211,267)	79.6 (70.9,87.2)	626 (570,688)	74.1 (67.5,80.7)	-6.9 (-12.5, -1.0)
**Viet Nam**	40697 (36757,44343)	102.3 (92.7,111.3)	74925 (68600,80667)	76.8 (70.7,82.3)	-24.9 (-28.7, -20.4)
**Southern Latin America**	24515 (22113,26843)	53.3 (48.1,58.2)	21618 (19466,23530)	25.8 (23.4,28.3)	-51.5 (-54.0, -49.2)
**Uruguay**	1445 (1292,1598)	39.5 (35.6,43.7)	1170 (1028,1292)	22.2 (19.7,24.5)	-43.8 (-47.5, -40.0)
**Argentina**	18262 (16412,20057)	57.6 (51.9,63.1)	14710 (13241,16125)	27.2 (24.5,29.8)	-52.8 (-55.7, -49.7)
**Chile**	4806 (4273,5302)	46.1 (40.9,50.7)	5737 (5111,6328)	23.6 (21.1,26.1)	-48.7 (-52.2, -45.2)
**Southern Sub-Saharan Africa**	15161 (13117,17201)	51.5 (44.7,58)	20705 (18235,23292)	36.6 (32.1,40.9)	-29.0 (-32.2, -25.7)
**Botswana**	454 (402,505)	79.8 (71.1,87.9)	880 (787,969)	59.6 (53.4,65)	-25.4 (-29.8, -21.3)

**Eswatini**	213 (190,234)	72.5 (65.3,79.3)	353 (319,383)	65.6 (59.8,70.7)	-9.6 (-13.9, -4.8)
**Lesotho**	537 (483,591)	65.3 (58.6,71.1)	880 (806,950)	86.6 (79.2,93.3)	32.5 (25.7, 40.2)
**Namibia**	518 (461,570)	81.5 (73.1,89.2)	747 (682,820)	55.9 (51.1,61.2)	-31.4 (-35.1, -27.1)
**South Africa**	12027 (10194,13799)	52.8 (45,60.3)	15401 (13262,17627)	34.3 (29.7,38.8)	-35.0 (-38.9, -31.1)
**Zimbabwe**	1412 (1262,1580)	32.3 (28.9,35.3)	2443 (2198,2702)	33.1 (30,36.1)	2.4 (-3.0, 8.9)
**Tropical Latin America**	52463 (44397,60679)	51.9 (43.4,59.8)	52688 (45343,60232)	20.8 (17.9,23.7)	-59.9 (-62.1, -57.4)
**Brazil**	51322 (43298,59534)	52 (43.3,60)	51212 (43934,58713)	20.7 (17.8,23.7)	-60.2 (-62.4, -57.6)
**Paraguay**	1141 (1052,1236)	47.2 (43.6,51)	1475 (1342,1616)	24.6 (22.3,26.8)	-47.9 (-51.2, -44.2)
**Western Europe**	127713 (113717,139441)	23.1 (20.7,25.2)	124279 (111584,135124)	13.2 (11.9,14.5)	-42.7 (-44.9, -40.7)
**Andorra**	8 (7,9)	14.1 (12.1,16.1)	16 (14,18)	11 (9.5,12.4)	-22.1 (-27.2, -16.1)
**Austria**	4363 (3821,4838)	37.3 (32.8,41)	2498 (2200,2804)	14.3 (12.6,16.1)	-61.6 (-64.4, -58.7)
**Belgium**	3559 (3165,3912)	24.3 (21.7,26.5)	3135 (2829,3428)	12.9 (11.8,14.2)	-46.6 (-50.4, -42.6)
**Cyprus**	265 (231,300)	33.3 (29.3,37.3)	357 (315,393)	18.7 (16.5,20.5)	-43.9 (-48.8, -38.7)
**Denmark**	1939 (1723,2122)	24.6 (22.1,26.9)	1500 (1313,1679)	13.1 (11.6,14.6)	-46.8 (-50.5, -42.6)
**Finland**	1604 (1467,1746)	23.3 (21.4,25.3)	2051 (1812,2305)	17 (15.2,18.9)	-26.9 (-32.0,

					-21.3)
**France**	14777 (13486,15956)	18.3 (16.7,19.7)	17253 (15444,18888)	11.9 (10.6,13.1)	-35.2 (-40.2, -30.8)
**Germany**	25899 (23132,28625)	21.5 (19.3,23.7)	24874 (22250,27565)	13.3 (11.9,14.9)	-37.9 (-44.1, -31.9)
**Greece**	6770 (6143,7346)	47.4 (43.2,51)	6577 (5911,7195)	25.7 (23.1,28)	-45.8 (-49.3, -42.3)
**Iceland**	68 (59,77)	23.7 (20.7,26.7)	64 (57,72)	11.4 (10,12.9)	-51.9 (-55.4, -47.8)
**Ireland**	934 (816,1049)	23.7 (20.9,26.2)	877 (770,985)	11.6 (10.3,13.1)	-50.8 (-55.0, -47.1)
**Israel**	1274 (1115,1424)	26.7 (23.6,29.6)	1565 (1381,1733)	12.5 (11.1,14)	-53.1 (-56.2, -49.2)
**Italy**	18650 (15356,21977)	22.6 (18.9,26.6)	19386 (17001,21929)	13.1 (11.6,14.6)	-42.1 (-47.0, -35.4)
**Luxembourg**	145 (133,157)	28.1 (25.7,30.3)	121 (110,133)	11.4 (10.2,12.5)	-59.5 (-61.9, -56.8)
**Malta**	121 (107,136)	29.5 (26.1,33)	142 (123,163)	15.2 (13.4,17.4)	-48.5 (-52.7, -43.4)
**Monaco**	14 (12,16)	21.4 (18.6,23.9)	12 (10,13)	12.7 (11.1,14.2)	-40.7 (-44.8, -36.3)
**Netherlands**	4201 (3806,4519)	21.5 (19.6,23.2)	4449 (3919,4966)	13.1 (11.6,14.6)	-39.2 (-43.6, -34.8)
**Norway**	1302 (1056,1564)	19.4 (15.9,22.9)	1223 (1042,1413)	12.3 (10.5,14.1)	-36.7 (-40.9, -30.9)
**Portugal**	6692 (6043,7312)	51.5 (46.8,55.7)	3922 (3570,4264)	15.7 (14.2,17.1)	-69.6 (-71.2, -67.5)
**San Marino**	6 (6,7)	18.6 (16.2,21)	9 (8,11)	12.3 (10.7,13.7)	-34.0 (-38.3, -29.4)
**Spain**	14458	28.5	13024	12.6 (11.3,14)	-55.6

	(12829,16016)	(25.4,31.2)	(11597,14241)		(-58.7, -52.5)
**Sweden**	2944 (2430,3493)	19.9 (16.6,23.3)	2873 (2392,3351)	13.8 (11.7,15.9)	-30.7 (-35.3, -25.7)
**Switzerland**	1877 (1690,2041)	18.2 (16.4,19.9)	1788 (1564,2010)	9.9 (8.6,11.2)	-45.8 (-50.5, -41.0)
**United Kingdom**	15737 (13293,18120)	18.4 (15.7,21)	16453 (14114,18774)	12.7 (11.1,14.3)	-31.2 (-34.8, -26.6)
**Western Sub-Saharan Africa**	82131 (72597,91400)	89.3 (78.5,99.8)	139009 (125319,152586)	65.3 (58.5,71.5)	-26.9 (-29.6, -24.0)
**Benin**	2105 (1900,2313)	96.3 (85.7,105.9)	4110 (3750,4529)	72.6 (65.3,79.8)	-24.6 (-29.1, -19.1)
**Burkina Faso**	3126 (2789,3430)	69.8 (62.3,76.2)	6357 (5774,6968)	63.4 (57,68.4)	-9.2 (-15.0, -2.3)
**Cabo Verde**	163 (144,181)	65.7 (58.3,72.8)	247 (220,268)	54.5 (48.1,59.8)	-17.0 (-22.0, -11.5)
**Cameroon**	4403 (3970,4828)	95.6 (85.9,104.8)	11010 (10105,12072)	80 (73.1,87.2)	-16.3 (-21.0, -10.7)
**Chad**	2764 (2508,3038)	91.8 (82.9,101)	5784 (5295,6293)	86.2 (78.5,93.9)	-6.1 (-11.3,0)
**Coted'Ivoire**	4427 (3992,4902)	92.8 (83.2,102)	9037 (8220,9804)	71.7 (65.1,78.3)	-22.7 (-27.1, -17.4)
**Gambia**	324 (289,361)	81.5 (72.9,89.9)	762 (688,837)	71.7 (64.6,78.7)	-12.0 (-17.0, -6)
**Ghana**	6743 (6128,7416)	95.8 (86.6,104.8)	14326 (13067,15797)	77.8 (70.6,85.2)	-18.8 (-23.6, -13.2)
**Guinea**	3282 (2943,3608)	95.9 (85.6,105.2)	5424 (4991,5903)	87.6 (80.2,95)	-8.7 (-13.9, -2.3)
**Guinea-Bissau**	541 (487,600)	122.4 (110.9,133.4)	776 (710,846)	92.8 (85.1,100.7)	-24.1 (-28.4, -19.8)

**Liberia**	1102 (1002,1212)	88.1 (79.2,96.4)	1548 (1398,1701)	63.5 (57.4,69.4)	-27.9 (-31.4, -23.6)
**Mali**	3502 (3155,3835)	88.9 (79.5,97.2)	6101 (5541,6717)	68.5 (61.7,74.4)	-23.0 (-27.3, -17.6)
**Mauritania**	943 (840,1049)	88.7 (78.8,97.9)	1268 (1134,1399)	56.4 (50.2,61.9)	-36.4 (-40.5, -31.7)
**Niger**	3099 (2800,3425)	97.7 (87.7,107.5)	6895 (6319,7565)	76.4 (69.1,82.7)	-21.8 (-26.5, -17.6)
**Nigeria**	39617 (33630,45908)	88.7 (74.2,102)	54201 (46891,61164)	56.4 (48.5,63.8)	-36.5 (-39.5, -32.5)
**Sao Tome and Principe**	58 (52,64)	86 (76.7,94.4)	87 (79,96)	72.1 (65.1,78.7)	-16.2 (-20.8, -11.0)
**Senegal**	2778 (2492,3077)	77.6 (69.1,85.8)	5077 (4610,5593)	61.9 (55.7,68.2)	-20.2 (-24.9, -15.1)
**Sierra Leone**	1878 (1697,2063)	82.7 (74.2,91.3)	3049 (2762,3326)	70.7 (64.1,76.9)	-14.5 (-18.8, -8.5)
**Togo**	1272 (1152,1405)	87.8 (78.9,96)	2947 (2663,3247)	71.9 (64.9,78.9)	-18.1 (-22.2, -13.5)

95% UI=95% uncertainty intervals. ASIR: Age-standardized inidence rate

Supplementary Table 3: Deaths of Intracerebral Hemorrhage in 1990 and 2021 and the percentage change in the age-standardised rates (ASRs) per 100,000, by location.

location	Number (95% UI) (1990)	ASR/100000 (95% UI) (1990)	Number (95% UI) (2021)	ASR/100 000 (95% UI) (2021)	Change ASR/100 000 (1990-2021) (95% UI)
**Global**	2341558 (2184645,2505961)	61.6 (57,66.1)	3308367 (3021075,3594725)	39.1 (35.6,42.5)	-36.6 (-42.5,-29.8)
**Andean Latin America**	7249 (6427,8328)	34.5 (30.6,39.7)	8766 (7271,10653)	14.9 (12.4,18.1	-56.8 (-65.4,-

				)	46.1)
**Bolivia (Plurinational State of)**	1925 (1436,2672)	59.6 (44.8,82.4)	2363 (1668,3270)	27.2 (19.4,37.4)	-54.3 (-64.9,-36.6)
**Ecuador**	1714 (1596,1841)	31.6 (29.2,34.1)	2177 (1742,2683)	13.7 (11,16.8)	-56.5 (-65.6,-45.4)
**Peru**	3610 (3055,4282)	29.4 (24.8,34.8)	4226 (3257,5548)	12.5 (9.6,16.4)	-57.5 (-69.3,-42.4)
**Australasia**	3192 (2950,3395)	13.7 (12.6,14.6)	3912 (3371,4301)	6.5 (5.7,7.2)	-52.3 (-56.4,-48.2)
**Australia**	2664 (2456,2855)	13.7 (12.6,14.7)	3289 (2827,3621)	6.5 (5.6,7.1)	-53 (-57.4,-48.6)
**New Zealand**	528 (488,559)	13.4 (12.4,14.2)	623 (534,685)	6.9 (5.9,7.5)	-48.8 (-53.7,-44.1)
**Caribbean**	12492 (11374,13523)	48.2 (44,52.2)	16729 (14312,19374)	31.2 (26.6,36.1)	-35.4 (-44.2,-25.1)
**Antigua and Barbuda**	32 (30,34)	58.3 (54,62.3)	31 (28,34)	30.6 (28,33.4)	-47.5 (-52.5,-41.8)
**Bahamas**	70 (64,77)	45.9 (41.7,50.3)	96 (78,118)	24.3 (19.9,29.5)	-47 (-58.3,-33.5)
**Barbados**	110 (102,119)	37.3 (34.6,40.2)	117 (94,144)	22.5 (18.1,27.7)	-39.6 (-53.8,-24.4)
**Belize**	37 (34,40)	38.9 (36.1,41.9)	73 (64,82)	25.4 (22.3,28.5)	-34.6 (-43.7,-25.5)
**Bermuda**	13 (11,15)	20.9 (18.6,24.1)	12 (10,14)	8.1 (6.9,9.7)	-61.2 (-69.1,-51.4)
**Cuba**	3093 (2874,3338)	30.6 (28.4,33)	3485 (3017,3947)	17.5 (15.2,19.9)	-42.7 (-51.4,-33.7)
**Dominica**	32 (29,35)	53.6 (48.1,59.3)	28 (24,34)	34.7 (29.9,41.1)	-35.3 (-46.3,-22.5)
**Dominican**	1648	46.1	3432	34.4	-25.3 (-

**Republic**	(1415,1897)	(39.4,53.1)	(2663,4402)	(26.7,44.1)	42.9,1.8)
**Grenada**	45 (40,50)	62.3 (55.5,69.6)	35 (31,39)	31.7 (27.8,35.2)	-49.1 (-57.7,-40.2)
**Guyana**	550 (497,608)	146.7 (133,161.7)	374 (287,475)	59.7 (46.2,75.2)	-59.3 (-69.4,-48.3)
**Haiti**	4339 (3319,5289)	137 (103.2,168.4)	5954 (4325,8006)	87.2 (61.9,117)	-36.4 (-52.7,-16.2)
**Jamaica**	859 (771,939)	47.6 (42.8,52)	1100 (860,1388)	35.1 (27.4,44.3)	-26.2 (-44.1,-3.4)
**Puerto Rico**	543 (512,571)	15.3 (14.4,16.1)	551 (451,643)	7.3 (6,8.5)	-52.6 (-60.9,-44.3)
**Saint Kitts and Nevis**	37 (34,42)	98.2 (89,109.9)	27 (23,32)	42.5 (36,49.1)	-56.7 (-64.8,-47.6)
**Saint Lucia**	57 (53,61)	68.4 (63.9,73.3)	70 (58,83)	29.5 (24.6,35)	-56.9 (-64.8,-48.5)
**Saint Vincent and the Grenadines**	45 (41,49)	63 (57.8,69.2)	48 (43,55)	34.9 (30.9,39.4)	-44.6 (-52.7,-35.7)
**Suriname**	168 (151,185)	67 (60.2,74.2)	287 (222,355)	45.7 (35.2,56.4)	-31.8 (-48.1,-13.3)
**Trinidad and Tobago**	366 (346,388)	45.9 (43.3,48.7)	417 (324,533)	21.7 (16.9,27.7)	-52.6 (-64,-39)
**United States Virgin Islands**	25 (21,30)	32.5 (26.7,38)	23 (18,29)	13.2 (10.7,16.5)	-59.3 (-68.8,-46.3)
**Central Asia**	32472 (30458,34622)	73.2 (68.1,78.4)	34963 (31599,38840)	47.9 (43.3,52.9)	-34.6 (-41.4,-27.5)
**Armenia**	982 (886,1080)	39.7 (35.6,43.9)	513 (454,575)	12 (10.6,13.4)	-69.8 (-74.2,-65)
**Azerbaijan**	3602 (3038,4184)	80.1 (68.3,92.9)	5025 (3923,6197)	60.9 (47.6,74.8)	-24 (-42.5,-2)

**Georgia**	6487 (5713,7252)	108.6 (95.6,121.8)	3388 (2969,3783)	54.3 (47.8,60.5)	-50 (-58.2,-41)
**Kazakhstan**	6674 (5818,7638)	56 (48.6,64)	8497 (7335,9767)	55.5 (48.6,63)	-0.9 (-17.6,17.7)
**Kyrgyzstan**	2244 (2020,2482)	78.3 (70.2,87)	1318 (1085,1549)	27.8 (23.2,32.4)	-64.5 (-70.7,-57.3)
**Mongolia**	1683 (1425,1982)	178.9 (151.2,211.3)	1926 (1590,2306)	98.1 (80.4,117.2)	-45.2 (-56.7,-30.1)
**Tajikistan**	1896 (1590,2220)	72.2 (60.3,84.9)	2600 (2024,3240)	54.1 (42.3,66.8)	-25.1 (-45.2,0.5)
**Turkmenistan**	947 (807,1138)	50.4 (42.7,61.1)	2488 (1874,3260)	62.6 (47.3,82.4)	24.1 (-5.6,57.1)
**Uzbekistan**	7957 (7312,8537)	70.7 (64.7,76.2)	9209 (7893,10710)	39.7 (34.4,45.9)	-43.9 (-52.8,-34)
**Central Europe**	78299 (75285,81136)	54.7 (52.5,56.7)	47995 (44202,51503)	21 (19.3,22.5)	-61.7 (-64.5,-58.6)
**Albania**	2387 (2111,2670)	143 (126.7,159.8)	4243 (3437,5072)	103.3 (84,123.3)	-27.7 (-42.3,-11.5)
**Bosnia and Herzegovina**	1191 (1002,1404)	32.4 (26.9,38.2)	1051 (811,1309)	16.6 (12.8,20.7)	-48.8 (-61.4,-33.5)
**Bulgaria**	12774 (12207,13337)	119.4 (113.8,124.2)	5562 (4774,6399)	39.4 (33.8,45.3)	-67 (-71.5,-62.1)
**Croatia**	2804 (2639,2962)	48.5 (45.8,51.2)	1371 (1196,1555)	14.4 (12.5,16.4)	-70.4 (-74.1,-66.1)
**Czechia**	4391 (4088,4699)	31.6 (29.4,33.8)	1775 (1558,1994)	7.8 (6.9,8.8)	-75.3 (-78.6,-71.9)
**Hungary**	7251 (6879,7604)	51.1 (48.3,53.6)	2139 (1833,2447)	10.7 (9.2,12.3)	-79 (-81.9,-75.8)
**Montenegro**	827 (725,931)	142.8 (125.9,160.4)	1369 (1172,1595)	159.3 (137.3,184.4)	11.5 (-6.2,32.5)

**North Macedonia**	1370 (1205,1553)	78.5 (69.2,88.9)	1502 (1214,1875)	52.8 (43.1,65.1)	-32.8 (-47.7,-13.9)
**Poland**	15854 (15150,16354)	37.7 (35.9,39)	8757 (7867,9547)	12 (10.9,13.1)	-68.1 (-70.8,-65.3)
**Romania**	18448 (16644,20140)	69.9 (62.7,76.4)	12915 (11433,14558)	33.6 (29.6,37.9)	-52 (-59,-44.3)
**Serbia**	6710 (5808,7739)	71.5 (61.8,82.6)	4832 (3972,5801)	28.3 (23.3,33.9)	-60.5 (-69.1,-50)
**Slovakia**	2482 (2168,2775)	41.8 (36.8,46.7)	1440 (1204,1743)	15.1 (12.6,18.3)	-63.9 (-70.4,-54.8)
**Slovenia**	558 (519,599)	22.5 (20.9,24.1)	342 (289,387)	6.7 (5.7,7.6)	-70.1 (-74.1,-65.9)
**Central Latin America**	21176 (20447,21805)	26.4 (25.2,27.3)	33528 (29703,37865)	13.7 (12.1,15.4)	-48.2 (-53.7,-42.2)
**Colombia**	5097 (4858,5299)	30.1 (28.6,31.4)	5046 (4215,5931)	9.1 (7.6,10.7)	-69.8 (-74.8,-64.6)
**Costa Rica**	279 (258,298)	16.4 (15.1,17.5)	424 (370,474)	7.7 (6.7,8.6)	-53 (-59.2,-47.1)
**El Salvador**	935 (841,1015)	30.7 (27.5,33.3)	859 (694,1049)	13.5 (10.9,16.5)	-56.1 (-65.7,-43.6)
**Guatemala**	1138 (1061,1220)	33.6 (30.8,36.7)	2218 (1898,2548)	21.3 (18.2,24.4)	-36.7 (-47,-26)
**Honduras**	914 (753,1078)	44.1 (36.2,52.8)	2926 (2338,3636)	50 (40,62.6)	13.4 (-9.7,44.5)
**Mexico**	8435 (8197,8633)	21.1 (20.4,21.7)	13568 (11953,15127)	11.1 (9.8,12.4)	-47.5 (-53.2,-41.6)
**Nicaragua**	439 (399,476)	28.1 (25.3,30.7)	635 (540,773)	13.6 (11.7,16.6)	-51.6 (-59.7,-40.5)
**Panama**	424 (394,454)	29 (26.9,31.2)	696 (540,838)	15.6 (12.1,18.8)	-46.3 (-58.1,-35.2)

**Venezuela (Bolivarian Republic of)**	3514 (3289,3744)	37.6 (34.9,40.2)	7156 (5347,9202)	24.4 (18.3,31.2)	-35 (-52.2,-16.4)
**Central Sub-Saharan Africa**	20780 (16341,26494)	98.9 (77.9,125.4)	36781 (27714,47047)	77.1 (58.6,99.5)	-22 (-38.5,-1)
**Angola**	4205 (3351,5285)	114.5 (91.4,142.1)	7358 (5576,9220)	71.1 (54.8,88.6)	-37.9 (-54.4,-17.7)
**Central African Republic**	1512 (1094,2042)	141.3 (104.2,189.4)	2276 (1582,3194)	115.4 (81,160.8)	-18.3 (-34.9,2.3)
**Congo**	1245 (970,1603)	126.5 (97.5,161.1)	1785 (1315,2327)	74.4 (57.1,92.4)	-41.2 (-54.1,-23.9)
**Democratic Republic of the Congo**	13076 (9776,17491)	89.9 (67.1,121.7)	24616 (17489,33334)	78.1 (55.8,106.2)	-13.1 (-35.5,16.3)
**Equatorial Guinea**	240 (180,310)	132 (101.4,169)	218 (138,321)	47.4 (31,67.9)	-64.1 (-75.7,-46.7)
**Gabon**	502 (376,633)	93 (69.7,117.9)	528 (380,713)	56.6 (41.9,74)	-39.2 (-53.6,-20.2)
**East Asia**	942386 (813480,1096840)	138.1 (120.2,159.9)	1366572 (1147252,1610948)	68.3 (57.4,80.2)	-50.5 (-59.9,-40.1)
**China**	913023 (784398,1064534)	139.7 (121.1,162)	1322893 (1108046,1567711)	68.8 (57.6,81.2)	-50.7 (-60.3,-39.9)
**Democratic People's Republic of Korea**	18703 (14347,23452)	137.4 (106.4,171.4)	37079 (29284,44919)	118.8 (94.3,144.2)	-13.5 (-32.4,14.6)
**Taiwan (Province of China)**	10660 (10287,11054)	75.2 (71.5,78)	6601 (5914,7132)	15.6 (14.1,16.8)	-79.2 (-80.9,-77.7)
**Belarus**	3214 (2823,3719)	25.1 (22.1,29.1)	3051 (2491,3654)	19.5 (15.9,23.3)	-22.6 (-40.2,-2.8)
**Estonia**	602 (556,650)	29.7 (27.4,32)	197 (172,223)	6.9 (6.1,7.8)	-76.6 (-79.7,-73.4)
**Latvia**	1680 (1544,1845)	47 (43.2,51.5)	625 (550,701)	15.2 (13.4,17)	-67.7 (-71.5,-

					63.1)
**Lithuania**	815 (749,886)	18.1 (16.7,19.8)	675 (588,749)	11.3 (9.8,12.6)	-37.8 (-45.5,-28.5)
**Republic of Moldova**	3593 (3320,3872)	87.9 (81.1,95.5)	1891 (1696,2105)	31.7 (28.4,35.2)	-64 (-68.3,-59)
**Russian Federation**	69607 (67233,70876)	40.7 (39.1,41.6)	57522 (53053,61872)	24.7 (22.8,26.6)	-39.3 (-43.7,-34.9)
**Ukraine**	19863 (17943,21784)	28 (25.2,30.6)	14298 (10820,18222)	19.1 (14.3,24.4)	-31.6 (-48.9,-11)
**Eastern Sub-Saharan Africa**	84394 (73095,97581)	119.8 (104.3,138.3)	111874 (95012,130095)	71.8 (60.8,83.4)	-40.1 (-49,-30.9)
**Burundi**	3868 (2953,4994)	168.4 (127.3,215.8)	3754 (2884,4657)	83.8 (64.1,103.7)	-50.3 (-63.6,-32.2)
**Comoros**	210 (160,266)	112.7 (88.3,142.5)	279 (214,351)	61.9 (48.1,77.6)	-45 (-58.9,-24.9)
**Djibouti**	123 (91,162)	101.6 (75.6,134.9)	360 (256,478)	64.2 (46.7,83.4)	-36.7 (-53.8,-12.5)
**Eritrea**	1742 (1349,2250)	166.8 (131.5,215.7)	2391 (1782,3197)	97.7 (74.1,128.2)	-41.4 (-53.2,-26)
**Ethiopia**	23884 (18987,30964)	128.9 (103.1,162.6)	20820 (16700,25525)	52 (41.3,64.2)	-59.7 (-71.1,-48.1)
**Kenya**	4676 (3464,5621)	62.5 (45.3,75.1)	11369 (8685,14322)	57.1 (43.5,71.9)	-8.5 (-25.5,13.4)
**Madagascar**	8268 (7204,9454)	168 (145.4,195.1)	13241 (9935,17160)	127.1 (93.3,162.6)	-24.4 (-42.9,-1.6)
**Malawi**	3678 (3092,4450)	101.3 (85.9,121)	5836 (4711,7044)	84.8 (67.9,102.7)	-16.3 (-33.7,4)
**Mozambique**	7182 (6074,8604)	127.7 (108.4,152.3)	13702 (10453,17639)	127.8 (97,163)	0.1 (-24.4,27.8)
**Rwanda**	5504 (4420,6800)	204 (165.6,251.2)	4288 (3112,5593)	77.8 (56.4,102)	-61.9 (-73.4,-

					48.4)
**Somalia**	3455 (2529,4551)	158.1 (119.2,201.9)	5868 (4086,8130)	106.4 (74.5,145.6)	-32.7 (-48.2,-11.5)
**South Sudan**	2884 (2213,3715)	115.6 (89.5,148.6)	2897 (2024,4046)	81.7 (59.3,109.9)	-29.3 (-47.3,-5)
**Uganda**	6323 (4765,8040)	104.2 (79.5,131.6)	8189 (5891,10928)	60.7 (43.4,80.3)	-41.8 (-57.3,-21.6)
**United Republic of Tanzania**	9181 (7609,11015)	90.2 (74.2,108.4)	12632 (9239,16781)	54.6 (39.5,71.7)	-39.4 (-53.8,-17.6)
**Zambia**	3355 (2763,4086)	131.2 (107.9,161.8)	6151 (4557,8062)	100.5 (75.3,128)	-23.4 (-42.8,0.9)
**High-income Asia Pacific**	60613 (56522,63819)	31.8 (29.5,33.5)	54899 (45364,60784)	10.2 (8.8,11)	-67.9 (-70.2,-65.9)
**Brunei Darussalam**	53 (45,62)	55.2 (46.3,64.6)	75 (64,89)	25.5 (21.6,29.8)	-53.7 (-62.9,-39.4)
**Japan**	35399 (32388,37051)	21.9 (19.8,23)	43254 (35296,47796)	10 (8.8,10.8)	-54.3 (-56.9,-52.2)
**Republic of Korea**	24519 (21445,26862)	102.6 (89.2,113.1)	11075 (9144,12771)	12.1 (10,14)	-88.2 (-89.8,-86)
**Singapore**	642 (610,670)	29.9 (28.3,31.4)	496 (446,537)	5.9 (5.3,6.4)	-80.2 (-82.1,-78.3)
**High-income North America**	39017 (36249,40680)	10.9 (10.2,11.4)	62750 (56014,66579)	9.3 (8.4,9.8)	-15.3 (-19.3,-12.1)
**Canada**	3141 (2915,3340)	9.6 (9,10.2)	4563 (3960,4946)	5.8 (5.1,6.3)	-39.5 (-44.4,-34.1)
**Greenland**	16 (14,18)	55.9 (48.1,63.9)	16 (13,18)	25.6 (21.2,30.9)	-54.3 (-62.3,-43.8)
**United States of America**	35859 (33193,37340)	11.1 (10.3,11.5)	58171 (52026,61737)	9.7 (8.7,10.3)	-12.6 (-16.9,-9.2)
**North Africa and Middle East**	96235 (84635,106834)	57.9 (50.4,65.2)	106099 (91137,121433)	25.4 (21.6,28.9)	-56.2 (-62,-49)
**Afghanistan**	8460	128.1	6844	68.9	-46.2 (-

	(6030,11138)	(91.2,169.4)	(4753,9390)	(46.1,95.6)	60.3,-29.1)
**Algeria**	5004 (3918,6210)	50.2 (38.1,63.1)	7091 (4831,10280)	24.6 (16.8,35.5)	-51 (-64.8,-37.1)
**Bahrain**	79 (71,87)	58 (52.1,64)	134 (112,158)	22.4 (19.2,26.3)	-61.4 (-68.5,-53.2)
**Egypt**	21025 (13622,27456)	82.4 (50.1,111.2)	17014 (10723,23174)	31.4 (19.8,42.8)	-61.9 (-70.4,-50.1)
**Iran (Islamic Republic of)**	5148 (4543,5662)	21.2 (18.3,23.4)	6875 (6193,7540)	9.5 (8.5,10.5)	-55.1 (-60.3,-48.3)
**Iraq**	5966 (4988,7152)	69.3 (57.2,83.5)	9865 (7601,12358)	44.5 (35,54.4)	-35.8 (-52.2,-14.5)
**Jordan**	554 (463,663)	38.4 (31.4,46.5)	935 (742,1136)	14.4 (11.5,17.6)	-62.5 (-71.5,-51.2)
**Kuwait**	88 (81,94)	13 (11.7,14.1)	213 (176,259)	7.8 (6.4,9.4)	-39.8 (-50.8,-28.1)
**Lebanon**	1148 (896,1449)	58.5 (45.6,73.4)	916 (750,1110)	14.2 (11.7,17.2)	-75.6 (-81.7,-67.6)
**Libya**	597 (464,760)	27.3 (20.2,36.4)	988 (666,1393)	19.5 (13.1,28)	-28.8 (-48.4,-3.3)
**Morocco**	7246 (5150,9500)	50.3 (34.4,66.9)	9573 (6557,12620)	30 (20.9,39.3)	-40.3 (-55.1,-24.7)
**Oman**	313 (233,409)	44.4 (33,59)	303 (237,374)	16.8 (13.4,20.2)	-62.2 (-72.8,-44.9)
**Palestine**	465 (374,562)	52.2 (41.9,63.4)	524 (444,607)	23.9 (20.3,27.8)	-54.1 (-64.3,-40.8)
**Qatar**	49 (41,58)	61.7 (51.2,72.1)	107 (81,140)	16.5 (13,20.4)	-73.3 (-80.1,-64.9)
**Saudi Arabia**	3519 (2736,4445)	60.1 (46.3,75.6)	5692 (4472,7076)	30 (24.8,36.2)	-50.1 (-62.7,-30.7)
**Sudan**	9042	88.5	6850	35	-60.5 (-

	(6705,11183)	(64.1,114.2)	(4598,9477)	(23.1,48.5)	70.9,-44.8)
**Syrian Arab Republic**	3771 (2981,4589)	62.6 (49.2,77.6)	4109 (3106,5277)	36.3 (28,45.4)	-42.1 (-58.4,-14.5)
**Tunisia**	1677 (1279,2115)	37.8 (27.9,48.6)	2320 (1417,3619)	18.9 (11.5,29.2)	-50 (-66.5,-29.8)
**Turkey**	16983 (14439,19543)	51.8 (43.3,60)	18325 (15013,22123)	21 (17.3,25.3)	-59.4 (-67.7,-47.5)
**United Arab Emirates**	175 (133,228)	40.4 (30.5,52.6)	491 (382,609)	22.4 (18.4,27)	-44.5 (-57.1,-29.1)
**Yemen**	4875 (3590,6335)	97.9 (70.6,132.9)	6830 (4715,9574)	51.6 (34.5,73.6)	-47.2 (-61.6,-25.4)
**Oceania**	3671 (2905,4578)	143.3 (116.3,176.7)	7262 (5783,8882)	110.4 (88.8,133.7)	-23 (-37.7,-3.8)
**American Samoa**	19 (17,21)	94.4 (84.5,104.4)	30 (26,36)	69.6 (59.4,82.1)	-26.3 (-38.2,-9.3)
**Cook Islands**	9 (8,11)	80.2 (69.6,91.4)	9 (8,11)	36.8 (29.8,44.1)	-54.1 (-63.8,-43)
**Fiji**	342 (294,398)	107.2 (92.8,124)	514 (400,646)	78.5 (63,96.9)	-26.8 (-42.8,-3.6)
**Guam**	30 (27,33)	46.6 (42.1,50.8)	48 (42,54)	22.9 (20.3,25.9)	-50.8 (-57.4,-42.1)
**Kiribati**	51 (41,59)	143.8 (117.1,167.8)	83 (66,103)	122.4 (100.9,148.1)	-14.9 (-31.4,7.7)
**Marshall Islands**	26 (22,31)	173.8 (145.6,205.2)	43 (33,56)	137.4 (105.4,175.4)	-21 (-36.7,-1.6)
**Micronesia (Federated States of)**	84 (67,102)	181.3 (146.5,219.8)	87 (68,110)	129.5 (101.8,162.9)	-28.6 (-43.7,-6.8)
**Nauru**	8 (6,10)	177.9 (142.8,223.1)	8 (7,11)	147.2 (117.7,183.5)	-17.3 (-34.9,8.9)
**Niue**	3 (2,3)	110.4 (92.1,	2 (1,2)	78.9	-28.6 (-

		131.6)		(65.3, 93.7)	42.4,-11.2)
**Northern Mariana Islands**	14 (11,18)	92.5 (77.4,112.9)	26 (23,29)	59.6 (51.7,66.1)	-35.5 (-49.8, -21.2)
**Palau**	10 (8,13)	109.6 (91,132.2)	16 (13,19)	77.3 (62.5,93.6)	-29.5 (-45.2, -8.6)
**Papua New Guinea**	2407 (1805,3153)	157.3 (119.6,204.6)	5228 (3843,6698)	122.1 (91.1,153.9)	-22.4 (-43.1,7)
**Samoa**	100 (82,117)	130.2 (107.6,151.2)	131 (109,158)	98.4 (82.7,117.9)	-24.4 (-37.7,-5.9)
**Solomon Islands**	211 (143,266)	186.8 (142.2,227.5)	479 (376,606)	160.7 (129.6,198.9)	-14 (-32.3,13.4)
**Tokelau**	2 (1,2)	131.4 (105.6,160.7)	1 (1,1)	75.8 (57.5,95.7)	-42.3 (-54.7,-27.8)
**Tonga**	28 (22,34)	55.4 (44.7,66.9)	34 (27,42)	44.2 (35.1,54.3)	-20.1 (-40,5.4)
**Tuvalu**	11 (9,13)	181.3 (149.9,213.5)	10 (9,13)	108 (91,128.6)	-40.5 (-51,-27.3)
**Vanuatu**	83 (67,105)	148.3 (121.6,183.9)	187 (150,228)	117.6 (95.3,141.6)	-20.7 (-35.5,-2.6)
**South Asia**	308097 (259917,356317)	56.4 (47.5,65.9)	558346 (456819,640417)	39.4 (32,45.3)	-30.2 (-39.9,-18.8)
**Bangladesh**	51427 (41841,61459)	111.5 (90.5,134)	87155 (67137,110040)	67.9 (51.6,85.1)	-39.1 (-52.3,-22.3)
**Bhutan**	122 (91,162)	53.7 (39.2,71.9)	171 (130,219)	29.5 (22.5,37.5)	-45.1 (-60.2,-25.7)
**India**	221736 (182032,259562)	49.9 (40.6,58.8)	411023 (332439,479199)	35.4 (28.4,41.5)	-29 (-40.8,-15.5)
**Nepal**	5777 (4482,7324)	65.1 (49.8,82.2)	8755 (6577,11471)	40 (29.4,52.2)	-38.6 (-54.8,-15.9)
**Pakistan**	29035	53.2	51242	44.3	-16.8 (-

	(23874,34579)	(42.9,63.8)	(41527,64735)	(35.9,55.6)	33.5,6.5)
**Southeast Asia**	270021 (241993,298671)	113.1 (99.8,126.5)	500812 (438999,571700)	80.7 (70.6,92.4)	-28.6 (-38.5,-17.7)
**Cambodia**	6243 (5302,7278)	151.2 (128.1,176.6)	11226 (8742,13978)	104.1 (81.9,127.3)	-31.1 (-47.5,-12.1)
**Indonesia**	115864 (98864,134424)	125.6 (103.6,150.8)	241504 (194265,298009)	112.6 (89.3,140.6)	-10.4 (-28.1,8)
**Lao People's Democratic Republic**	3639 (2844,4608)	177.1 (136.8,221.4)	4316 (3309,5384)	99.8 (77,121.9)	-43.6 (-58.2,-25)
**Malaysia**	7280 (6578,7930)	81.9 (74,89.6)	12826 (11480,14251)	48.3 (43.2,54)	-41 (-48.9,-32)
**Maldives**	117 (101,135)	138.7 (121.2,156.7)	115 (93,137)	37.3 (30.5,44.4)	-73.1 (-79.1,-66.4)
**Mauritius**	519 (490,552)	71.7 (67.7,76)	472 (437,497)	26.5 (24.6,27.8)	-63.1 (-65.9,-60.7)
**Myanmar**	40178 (31922,49969)	178.2 (144,221)	46968 (37644,58912)	101.5 (81.4,126.8)	-43 (-57.3,-22.3)
**Philippines**	19915 (18187,21566)	73.6 (67.3,79.5)	51846 (43593,60218)	64.6 (54.6,74.8)	-12.2 (-25.8,3)
**Seychelles**	36 (31,42)	63.3 (55.4,74.1)	38 (33,44)	34.1 (29.3,39.8)	-46.2 (-53.8,-36.9)
**Sri Lanka**	6463 (5722,7182)	69.4 (61.3,76.7)	9520 (6512,12873)	38.2 (26.2,51.3)	-45 (-63.6,-22.9)
**Thailand**	19492 (16685,22181)	61 (51.6,70.3)	32814 (25457,40801)	31 (24.2,38.6)	-49.2 (-61.5,-32.6)
**Timor-Leste**	302 (246,366)	117.3 (93.3,144.1)	795 (587,1022)	98.1 (72.6,126.7)	-16.3 (-38.5,11.3)
**Viet Nam**	49582 (39145,62838)	130.6 (102.4,164.4)	87674 (70631,104639)	95.4 (76.9,113)	-26.9 (-45.7,-4.8)
**Southern Latin**	18951	41.7 (39.4,44)	13214	15	-64.1 (-

**America**	(17972,19995)		(12212,14024)	(13.9,15.9)	66.9,-61.5)
**Uruguay**	1264 (1181,1344)	32.8 (30.7,34.9)	822 (746,881)	14 (12.8,15)	-57.3 (-61.5,-53.2)
**Argentina**	14350 (13527,15309)	45.3 (42.5,48.4)	9371 (8670,9988)	16.6 (15.3,17.7)	-63.4 (-66.6,-60.2)
**Chile**	3337 (3172,3516)	33.9 (32.2,35.7)	3021 (2762,3257)	11.7 (10.7,12.6)	-65.6 (-68.3,-63)
**Southern Sub-Saharan Africa**	13088 (11383,14453)	49.3 (42.3,55)	27476 (25442,29751)	50.7 (46.8,55.1)	2.8 (-8,19.3)
**Botswana**	501 (380,632)	100.3 (77,126.6)	618 (468,817)	48 (37.2,62.5)	-52.2 (-65.2,-34.4)
**Eswatini**	263 (211,322)	102.7 (81.7,126.4)	434 (291,622)	84.2 (58.6,117.2)	-18.1 (-41.5,14)
**Lesotho**	692 (544,866)	88.5 (69.1,110.7)	1205 (892,1608)	120.2 (91.1,157.5)	35.7 (-2.4,96.8)
**Namibia**	582 (481,701)	99.8 (81.6,122.7)	892 (664,1140)	72.5 (54.4,91.6)	-27.3 (-45.6,-5.2)
**South Africa**	8968 (7485,10167)	42.9 (35,49.2)	19000 (17069,20756)	43.8 (39.2,47.9)	1.9 (-9.1,19.1)
**Zimbabwe**	2082 (1702,2460)	58.3 (47.8,69)	5327 (4156,6695)	83.3 (66.8,100.9)	42.7 (9.2,91.6)
**Tropical Latin America**	44826 (43213,46160)	48.5 (46.3,50.2)	43863 (41028,45959)	17.1 (16,17.9)	-64.7 (-66.5,-63.1)
**Brazil**	43826 (42285,45163)	48.5 (46.3,50.2)	42419 (39721,44402)	16.9 (15.8,17.8)	-65.1 (-66.8,-63.4)
**Paraguay**	1000 (876,1138)	45.7 (39.9,52)	1444 (1100,1820)	25.2 (19.3,31.6)	-44.9 (-58.7,-28.1)
**Western Europe**	110336 (103441,115605)	18.7 (17.5,19.5)	88732 (76162,95377)	8.1 (7.1,8.6)	-56.9 (-59.8,-54.7)

**Andorra**	5 (3,6)	8.9 (6.4,11.9)	8 (6,11)	5.1 (3.7,6.7)	-42.7 (-61.7,-18.3)
**Austria**	2105 (1946,2247)	17.2 (16,18.3)	1251 (1078,1369)	5.9 (5.2,6.4)	-65.5 (-68.7,-62.6)
**Belgium**	3126 (2890,3353)	20 (18.5,21.4)	2347 (1987,2589)	8.6 (7.5,9.4)	-56.9 (-61,-53)
**Cyprus**	256 (215,304)	42.8 (36,50.2)	211 (172,247)	11.5 (9.5,13.4)	-73.1 (-78.7,-65.8)
**Denmark**	1683 (1556,1791)	19.8 (18.5,21.1)	1207 (1070,1311)	9.1 (8.1,9.9)	-54 (-57.8,-50.3)
**Finland**	1114 (1003,1239)	15.4 (13.9,17)	1125 (957,1234)	7.7 (6.7,8.4)	-49.9 (-55.9,-44.2)
**France**	14257 (13028,15133)	16.6 (15.2,17.6)	11601 (9953,12685)	6.6 (5.8,7.2)	-60.2 (-63.6,-57.1)
**Germany**	20913 (18670,23651)	16.1 (14.4,18)	14801 (12749,16158)	6.7 (5.9,7.2)	-58.3 (-63.7,-53.2)
**Greece**	7137 (6659,7516)	48.4 (44.8,51)	5589 (4894,6038)	19.1 (17,20.4)	-60.5 (-63,-57.9)
**Iceland**	41 (38,44)	13.8 (12.7,14.8)	38 (31,42)	5.8 (4.9,6.4)	-58.1 (-62.8,-53.2)
**Ireland**	609 (569,641)	15 (13.9,15.8)	426 (365,471)	5.1 (4.3,5.6)	-66.3 (-69.8,-62.9)
**Israel**	1146 (1061,1220)	24 (22.1,25.5)	1077 (941,1181)	8.1 (7.2,8.9)	-66.2 (-69.7,-63.2)
**Italy**	16789 (15587,17543)	18.9 (17.5,19.7)	17121 (14326,18658)	9.6 (8.3,10.3)	-49.1 (-52.7,-46.4)
**Luxembourg**	154 (145,162)	28.4 (26.8,29.8)	103 (90,114)	8.8 (7.7,9.7)	-69 (-72.6,-65.5)
**Malta**	98 (92,104)	23.6 (22,25)	77 (66,86)	7.3 (6.3,8.1)	-69.2 (-72.5,-65.5)
**Monaco**	16 (12,20)	21.4 (16.4,26)	10 (8,12)	9 (7,11.3)	-57.7 (-

					68.8,-39.8)
**Netherlands**	3088 (2838,3282)	15.1 (13.9,16)	2985 (2573,3255)	7.6 (6.7,8.3)	-49.4 (-53.6,-45.4)
**Norway**	952 (883,996)	13 (12.1,13.6)	772 (664,835)	6.7 (5.8,7.2)	-48.6 (-52.3,-45.6)
**Portugal**	7530 (7072,7885)	55.9 (52.3,58.4)	3708 (3260,4023)	13.2 (11.9,14.3)	-76.3 (-78.2,-74.5)
**San Marino**	6 (5,7)	16.3 (13.4,19.2)	5 (3,7)	5.7 (3.7,8)	-65 (-76.5,-49.8)
**Spain**	13158 (12131,13881)	24.4 (22.5,25.7)	9634 (8086,10698)	8.1 (7,8.9)	-66.6 (-69.3,-64)
**Sweden**	2198 (2035,2334)	13.5 (12.5,14.3)	1791 (1530,1998)	6.9 (6,7.7)	-48.6 (-54.2,-43)
**Switzerland**	1438 (1279,1595)	12.9 (11.6,14.3)	941 (773,1049)	4.3 (3.6,4.7)	-67 (-71,-62.7)
**United Kingdom**	12425 (11752,12779)	13.4 (12.7,13.8)	11827 (10455,12573)	8 (7.2,8.5)	-40.1 (-43.8,-37.8)
**Western Sub-Saharan Africa**	74890 (60442,91578)	89 (71.6,109)	105535 (83668,126793)	56.7 (44.4,67.2)	-36.3 (-45.8,-25.1)
**Benin**	1987 (1648,2429)	103.4 (85.5,127.2)	3334 (2561,4094)	70.6 (54.4,87.6)	-31.7 (-46,-12.7)
**Burkina Faso**	2863 (2223,3545)	69.8 (54.2,85.6)	5043 (3749,6349)	57.9 (43,73.6)	-17 (-35.1,6.8)
**Cabo Verde**	109 (88,129)	46.3 (37.4,55.3)	162 (127,202)	37.6 (29.2,46.7)	-18.9 (-38.4,7.8)
**Cameroon**	3873 (2904,5149)	92.7 (67.8,121.3)	8897 (6143,12323)	75.1 (52.1,101.8)	-19 (-38.8,12.2)
**Chad**	2361 (1869,2891)	84.1 (65.4,103.1)	4626 (3403,6195)	80.5 (58.7,106.7)	-4.3 (-28.4,24.2)
**Coted'Ivoire**	3077 (2370,4067)	78 (60.9,101.6)	6644 (4768,8746)	61.9 (45.1,81.3)	-20.7 (-41.3,8.1)
**Gambia**	281 (216,361)	82.7	725 (548,930)	77.6	-6.2 (-

		(63.7,106.8)		(58.4,99.6)	30,27.3)
**Ghana**	6360 (5016,8050)	103.9 (80,132.1)	12668 (9411,16306)	80 (58.6,102.6)	-23 (-42.3,4.8)
**Guinea**	2962 (2332,3735)	89.4 (69.7,114.7)	4199 (3048,5404)	76.3 (55.8,97.4)	-14.7 (-36.3,16.6)
**Guinea-Bissau**	590 (450,776)	147.5 (112.5,191.2)	789 (590,1009)	113.9 (85.7,145.4)	-22.8 (-42,-0.6)
**Liberia**	1002 (792,1242)	85.6 (68.4,104.8)	1427 (1042,1911)	70.2 (51.9,93.3)	-18 (-39.2,11.9)
**Mali**	3306 (2511,4206)	86 (63.7,110.4)	5048 (3674,6526)	58.9 (42.8,76)	-31.5 (-46,-12.4)
**Mauritania**	974 (773,1244)	101.3 (79,131.2)	1148 (846,1560)	56.2 (41.5,76.3)	-44.5 (-58.8,-22.6)
**Niger**	2514 (1834,3426)	92.4 (67.3,124.4)	5783 (3929,7888)	76 (51.9,103)	-17.8 (-35.6,5.8)
**Nigeria**	37012 (27804,48005)	89 (66.8,116.9)	34626 (26515,44352)	40.7 (31.6,51.1)	-54.2 (-64.2,-39.1)
**Sao Tome and Principe**	42 (35,49)	67 (55.7,77.9)	58 (48,73)	55 (45.4,66.3)	-17.8 (-34,4.9)
**Senegal**	2715 (2208,3353)	83.9 (67.9,103.8)	4873 (3660,6244)	66.5 (49.6,84.9)	-20.7 (-38.7,1.5)
**Sierra Leone**	1782 (1425,2267)	83.5 (67,104.5)	2675 (1975,3562)	71.2 (53.8,94.3)	-14.8 (-33.8,11)
**Togo**	1079 (887,1310)	88.6 (73.6,108.5)	2807 (2029,3690)	79.5 (59.1,102.7)	-10.3 (-32.2,17.7)

95% UI=95% uncertainty intervals. ASDR: Age-standardized death rate

Supplementary Table 4:DALYs of Intracerebral Hemorrhage in 1990 and 2021 and the percentage change in the age-standardised rates (ASRs) per 100,000, by location.

location	Number (95% UI) (1990)	ASR/ 100000 (95% UI) (1990)	Number (95% UI) (2021)	ASR/ 100000 (95% UI) (2021)	Change ASR/ 100000 (1990-2021) (95% UI)
**Global**	63197955 (59216767,67076895)	1516.8 (1421,1613.3)	79457427 (72748913, 85480165)	923.6 (844.8,993.2)	-39.1 (-44.7,-33.1)
**Andean Latin America**	228425 (200623,263937)	927.4 (820.8,1065.1)	234343 (193965,284558)	381.9 (316.4,463.7)	-58.8 (-67,-48.8)
**Bolivia (Plurinational State of)**	61298 (45919,85399)	1558.4 (1167.9,2159.4)	62243 (44498,84686)	647.1 (463,881.2)	-58.5 (-69.2,-42.3)
**Ecuador**	54226 (50814,57554)	857.7 (803,914.6)	54638 (43759,67963)	328.1 (263.1,407.8)	-61.8 (-70.1,-52.1)
**Peru**	112901 (96605,133332)	786.7 (667.8,929.4)	117462 (90446,150853)	336.4 (258.9,433.3)	-57.2 (-69.1,-42.3)
**Australasia**	68019 (63608,72028)	290.8 (271.6,308.2)	67763 (61317,73378)	126.6 (115.9,136.7)	-56.5 (-59.8,-53.1)
**Australia**	56337 (52564,60099)	289 (269.7,307.8)	56597 (51170,61414)	125.2 (114.4,135.7)	-56.7 (-59.9,-53.1)
**New Zealand**	11682 (10881,12341)	300.4 (279.9,317)	11167 (10099,12080)	133.8 (121.9,144.3)	-55.5 (-59.1,-51.8)
**Caribbean**	353990 (319027,388722)	1280.1 (1161.5,1401.8)	443350 (375380,519204)	839.6 (709.9,983.2)	-34.4 (-43.9,-23.1)
**Antigua and Barbuda**	716 (668,764)	1366.9 (1274,1455.6)	721 (662,790)	666.5 (613.6,726.3)	-51.2 (-56.1,-45.9)
**Bahamas**	1978 (1797,2160)	1170.5 (1062.5,1279.7)	2649 (2139,3268)	615.2 (499.3,754.4)	-47.4 (-58.3,-33)
**Barbados**	2400	875.2	2548	509.8	-41.8 (-

	(2243,2576)	(819.5,935.9)	(2021,3171)	(403.6,634.6)	56.1,-27.3)
**Belize**	969 (908,1039)	955.2 (894.4,1024.8)	1936 (1698,2176)	604.9 (531.9,679.9)	-36.7 (-45.6,-27.6)
**Bermuda**	312 (280,355)	493.2 (442.8,561.2)	240 (202,286)	187.9 (158.1,223.4)	-61.9 (-69.3,-53)
**Cuba**	77961 (72922,82980)	755.7 (706.7,804.4)	77947 (67022,88956)	417.1 (359.2,475.1)	-44.8 (-53,-36.4)
**Dominica**	705 (640,770)	1191.4 (1081.7,1302.9)	659 (552,799)	783.2 (657.6,945.1)	-34.3 (-45.9,-20.6)
**Dominican Republic**	48808 (42317,55991)	1150 (991.8,1321.9)	92540 (72337,119199)	896.9 (701.7,1153.7)	-22 (-40.8,5.1)
**Grenada**	1092 (981,1219)	1618 (1454.4,1803.2)	871 (750,979)	740.7 (643.2,831.8)	-54.2 (-62.1,-45.7)
**Guyana**	15827 (14250,17541)	3775.8 (3395.2,4177.3)	10348 (7879,13297)	1503.9 (1152.9,1922.2)	-60.2 (-70.4,-48.7)
**Haiti**	138609 (107737,173837)	3553.3 (2729.3,4324.7)	179645 (129578,242449)	2169.3 (1589.4,2920.3)	-38.9 (-55,-18.2)
**Jamaica**	21102 (19200,22994)	1190.6 (1085.7,1293.2)	25032 (19310,31761)	812.4 (626,1031.5)	-31.8 (-48.6,-11.4)
**Puerto Rico**	13171 (12540,13827)	365.5 (347.4,383.3)	11227 (9324,13092)	177.4 (146.1,207.3)	-51.5 (-59.8,-43.3)
**Saint Kitts and Nevis**	861 (790,958)	2384.7 (2189.8,2634.1)	708 (579,848)	972.6 (806.7,1149.2)	-59.2 (-66.9,-50.4)
**Saint Lucia**	1422 (1340,1516)	1607.6 (1511.9,1715.4)	1641 (1350,1967)	684 (563.9,817.8)	-57.4 (-65.5,-48.6)
**Saint Vincent and the Grenadines**	1076 (995,1171)	1458.1 (1346.3,1589.6)	1186 (1036,1350)	835.8 (732.8,948.6)	-42.7 (-51,-33.1)
**Suriname**	4657	1697.2	7620	1170.4	-31 (-

	(4066,5124)	(1506.9,1866.9)	(5979,9331)	(921.9,1426.8)	47.3,-12.7)
**Trinidad and Tobago**	9642 (9134,10201)	1113.8 (1053.5,1178.8)	10306 (7905,13216)	541 (415.8,693.9)	-51.4 (-63.3,-37)
**United States Virgin Islands**	696 (566,822)	775.3 (628.7,905)	522 (415,653)	329.3 (262.3,409.5)	-57.5 (-68,-43.2)
**Central Asia**	837737 (792642,890168)	1735.1 (1635.1,1846.4)	884451 (792892,985113)	1066.6 (961.2,1184.2)	-38.5 (-44.8,-31.9)
**Armenia**	23743 (21648,25943)	868.4 (791,948.8)	11833 (10559,13317)	281.7 (251.4,316.8)	-67.6 (-72,-62.5)
**Azerbaijan**	90542 (75742,106432)	1782.7 (1487.3,2096.6)	115226 (89453,142874)	1197.6 (933.8,1478.1)	-32.8 (-50.1,-12.7)
**Georgia**	151086 (133221,168028)	2433 (2144.4,2711.2)	67427 (58933,76196)	1156.9 (1003.7,1318.3)	-52.4 (-60,-43.7)
**Kazakhstan**	176952 (156314,200157)	1356.8 (1197.3,1538.3)	206921 (176004,239608)	1172.4 (1009.2,1345.7)	-13.6 (-27.8,2.5)
**Kyrgyzstan**	60451 (55154,66486)	1964 (1786.6,2161.3)	39214 (32283,46177)	728.9 (603.3,853.8)	-62.9 (-69.5,-55.2)
**Mongolia**	42516 (35919,49959)	3984.2 (3373.2,4688.6)	52403 (43264,62909)	2174.5 (1802.3,2585.3)	-45.4 (-57.3,-30.2)
**Tajikistan**	50767 (43113,59011)	1734.6 (1467.7,2020.7)	69630 (54485,87307)	1168.5 (913.5,1455.6)	-32.6 (-50.5,-9.6)
**Turkmenistan**	28459 (24524,33447)	1350.2 (1162.4,1599.4)	73336 (55721,94681)	1651.3 (1254.7,2137.7)	22.3 (-7.1,55.4)
**Uzbekistan**	213220 (198749,226907)	1750.6 (1627,1867.6)	248461 (211433,290713)	912 (786.4,1059.8)	-47.9 (-56.2,-38)
**Central Europe**	1853094 (1792178,1915611)	1254.4 (1211.8,1296.6)	957740 (882029,1029987)	457.1 (419.7,492.7)	-63.6 (-66.3,-60.6)
**Albania**	48263 (42934,54062)	2509.4 (2235.6,281	65219 (53094,78550)	1552.4 (1261.5,187	-38.1 (-51.5,-

		5.1)		0)	23.6)
**Bosnia and Herzegovina**	30421 (25778,35683)	732.8 (621.2,858.9)	20977 (16131,26391)	348.6 (268,435.4)	-52.4 (-64.3,-38)
**Bulgaria**	296285 (283778,309810)	2528.3 (2423.2,2641.5)	114715 (98095,133746)	892.5 (759.1,1044.1)	-64.7 (-69.9,-58.5)
**Croatia**	68661 (64635,72866)	1125.6 (1062.6,1190.2)	25824 (22646,29404)	299.3 (262.3,340.4)	-73.4 (-76.9,-69.3)
**Czechia**	101376 (94126,108983)	745.6 (691.8,801.1)	35133 (30940,39726)	172.7 (151.8,196.5)	-76.8 (-79.8,-73.6)
**Hungary**	165400 (157443,173752)	1171.8 (1116,1231.9)	45991 (39477,52795)	261.1 (224.1,299.7)	-77.7 (-80.8,-74.5)
**Montenegro**	16551 (14531,18592)	2719.1 (2388.3,3045.4)	22949 (19574,27078)	2510.1 (2158.9,2941)	-7.7 (-24,10)
**North Macedonia**	33424 (29466,37527)	1778.2 (1569.1,1999.5)	32191 (25402,40294)	1018.4 (813.5,1260.2)	-42.7 (-55.3,-25.9)
**Poland**	381328 (366793,393002)	890.1 (855.5,916.8)	185170 (168955,201869)	282.1 (257.9,308.3)	-68.3 (-71,-65.2)
**Romania**	447147 (406147,485511)	1608.9 (1468.2,1746)	264153 (232990,297279)	755.5 (664.8,852.5)	-53 (-60,-45.9)
**Serbia**	161456 (140079,185217)	1524.8 (1330.9,1746.1)	94196 (77373,112748)	585.3 (483.5,701.4)	-61.6 (-69.8,-51.4)
**Slovakia**	59401 (52062,66277)	1001.1 (878.5,1115.2)	31471 (26505,37983)	344.7 (289.8,415.4)	-65.6 (-71.7,-56.6)
**Slovenia**	13745 (12736,14739)	559.4 (519.5,599.9)	5812 (5000,6549)	129.7 (111.8,146.5)	-76.8 (-79.8,-73.6)
**Central Latin America**	617861 (601144,634932)	645.7 (625.5,664.1)	853699 (762557,971209)	335.5 (299.9,381.5)	-48 (-53.8,-41.5)
**Colombia**	143842 (138221,149209)	734.8 (702.9,763.9)	118100 (98664,139123)	215.1 (180.1,253.3)	-70.7 (-75.7,-65.8)
**Costa Rica**	7191	386.6	10216	186.5	-51.8 (-

	(6740,7622)	(361.6,410.5)	(8998,11349)	(164.4,207)	57.7,-45.8)
**El Salvador**	28081 (25485,30559)	813.3 (738.7,886.1)	21001 (16889,25704)	339 (271.4,416.1)	-58.3 (-67.5,-46.7)
**Guatemala**	39046 (37066,41397)	873.4 (817.2,934)	58589 (50234,67434)	498.6 (427.3,573.6)	-42.9 (-52.4,-33.5)
**Honduras**	29507 (24789,34654)	1151.6 (962.7,1354.2)	73226 (59082,91180)	1110.4 (898,1370.4)	-3.6 (-23.9,24.7)
**Mexico**	246006 (240210,252666)	501 (489.2,513.6)	356817 (316088,400701)	275.9 (244.3,309.2)	-44.9 (-51,-38.5)
**Nicaragua**	14175 (12979,15437)	712.5 (644.9,771.4)	16900 (14386,20430)	327.9 (279.8,397.4)	-54 (-61.5,-43.3)
**Panama**	11009 (10346,11674)	692.2 (648.9,735.1)	16320 (12737,19642)	369.2 (288.1,444.4)	-46.7 (-58,-35.8)
**Venezuela (Bolivarian Republic of)**	99004 (93538,104384)	916.4 (862,970.4)	182530 (135007,237172)	601 (445.9,777.4)	-34.4 (-51.6,-15.8)
**Central Sub-Saharan Africa**	676632 (518834,885183)	2422.3 (1913.2,3077.3)	1064701 (797681,1357572)	1772.6 (1344.9,2260.3)	-26.8 (-42.6,-6.4)
**Angola**	143679 (113130,185092)	2848.9 (2282.6,3571.2)	219652 (168036,276356)	1651.5 (1258,2061.5)	-42 (-57.9,-22.8)
**Central African Republic**	49090 (34955,68414)	3542.2 (2594.4,4768.3)	72279 (49271,103092)	2799.7 (1966.1,3899)	-21 (-39.3,0.4)
**Congo**	36927 (28397,48019)	3087.7 (2406.3,3981.5)	52248 (38140,69644)	1744.5 (1296.5,2262.2)	-43.5 (-57.1,-26.4)
**Democratic Republic of the Congo**	426556 (311770,577050)	2185 (1642.5,2913.5)	699729 (495548,941354)	1775.2 (1278.6,2390.9)	-18.8 (-39.4,8.9)
**Equatorial Guinea**	7324 (5388,9530)	3257.9 (2454.5,4194.2)	6504 (4025,9705)	1101 (709.7,1618.9)	-66.2 (-77.5,-49.4)

**Gabon**	13056 (9771,16349)	2183.8 (1639.5,2741.7)	14289 (9847,19672)	1292.2 (928.6,1747.8)	-40.8 (-55.4,-20.4)
**East Asia**	23549106 (20369697,27334077)	2810.1 (2431.5,3246.3)	28529140 (23808304,33766175)	1353.1 (1138.3,1594)	-51.8 (-60.6,-41)
**China**	22779117 (19630525,26510841)	2830 (2441.8,3281.1)	27463746 (22839243,32676709)	1351.6 (1129.1,1600.9)	-52.2 (-61.2,-41.1)
**Democratic People's Republic of Korea**	493967 (370680,630250)	3038 (2335.4,3822.3)	907833 (708135,1120909)	2745.1 (2159.7,3385.5)	-9.6 (-31.2,20.7)
**Taiwan (Province of China)**	276022 (267159,285203)	1703.1 (1645.5,1761.9)	157562 (144186,169334)	402.4 (369,430.9)	-76.4 (-78.2,-74.7)
**Belarus**	90994 (81402,104437)	718.2 (642,825.7)	78410 (63989,94812)	527.1 (429.5,637.4)	-26.6 (-43,-8.6)
**Estonia**	14507 (13350,15658)	720.1 (661.8,776.9)	3988 (3518,4494)	166.5 (146,187.4)	-76.9 (-80.1,-73.7)
**Latvia**	37922 (34930,41476)	1069.9 (987.3,1170.4)	12811 (11287,14340)	361 (316.8,405.7)	-66.3 (-70.4,-61.7)
**Lithuania**	21994 (20214,23861)	496.8 (456.4,538.6)	14124 (12417,15724)	273.8 (241.2,304.4)	-44.9 (-51.7,-37.4)
**Republic of Moldova**	90728 (84031,97226)	2043.7 (1893.1,2194.5)	45113 (40383,50633)	776 (694.2,870.8)	-62 (-66.8,-57)
**Russian Federation**	1726577 (1685997,1756240)	966.6 (942.1,984.1)	1393904 (1291389,1501590)	628.6 (582.1,677.1)	-35 (-39.9,-29.9)
**Ukraine**	519074 (471815,564758)	736.5 (669.5,799.5)	381988 (282138,487859)	547.9 (404.4,699.7)	-25.6 (-45,-3)
**Eastern Sub-Saharan Africa**	2529830 (2156354,2987079)	2919.9 (2533.1,3377.8)	3231776 (2741932,3779885)	1694.9 (1441.6,1964.7)	-42 (-51.8,-32.7)
**Burundi**	115001 (87632,150369)	4258.5 (3211.8,5544.4)	111540 (86083,138710)	1986.1 (1533.2,2458.6)	-53.4 (-66.5,-35)

**Comoros**	6298 (4494,8106)	2758.7 (2085.1,3492.3)	7392 (5666,9299)	1433.6 (1099.3,1795.6)	-48 (-62,-27.9)
**Djibouti**	3924 (2937,5138)	2416 (1802.3,3162.2)	10687 (7565,14433)	1500.1 (1080.8,1974.7)	-37.9 (-55.8,-12.2)
**Eritrea**	58817 (45179,76658)	4218 (3300.6,5443.9)	72883 (52892,97728)	2306.2 (1739.4,3055)	-45.3 (-57.3,-29.3)
**Ethiopia**	729920 (572900,977208)	3180.3 (2535.1,4124.6)	568468 (458477,688994)	1186.1 (956.8,1451.5)	-62.7 (-74.4,-51.5)
**Kenya**	128881 (100219,152626)	1406.3 (1047.9,1691.1)	309639 (239190,391183)	1266.4 (973.6,1593.1)	-9.9 (-26.7,11.7)
**Madagascar**	253378 (219507,287963)	4240 (3690.6,4843.4)	421853 (315226,545905)	3110.4 (2339.9,4011)	-26.6 (-45,-3.7)
**Malawi**	113696 (93073,142743)	2447.1 (2055.9,2941.6)	170118 (137142,206259)	2017.3 (1622,2434.2)	-17.6 (-34.6,3.7)
**Mozambique**	206272 (172194,250241)	3093.3 (2622.3,3688.7)	408783 (306981,536142)	3195.8 (2453.6,4107.9)	3.3 (-22.2,32.8)
**Rwanda**	168709 (134226,209190)	5105.7 (4120,6308)	116450 (84177,152026)	1732.9 (1261,2263.6)	-66.1 (-76.9,-53.1)
**Somalia**	113911 (82748,154447)	3876.3 (2875.4,5059)	185015 (124189,261150)	2542.1 (1788,3487)	-34.4 (-51.2,-11.2)
**South Sudan**	82590 (62627,107051)	2791.4 (2120,3614)	88306 (60388,124037)	1947.4 (1370,2702.3)	-30.2 (-49.1,-3.6)
**Uganda**	183172 (139103,234338)	2441.4 (1844.3,3094.3)	237353 (175517,316183)	1405.8 (1016.9,1869.3)	-42.4 (-58.4,-22.4)
**United Republic of Tanzania**	266107 (222413,318596)	2139.7 (1777.5,2567.8)	344582 (251824,452496)	1234.4 (904.8,1637.1)	-42.3 (-56.7,-21)
**Zambia**	97346 (80343,118630)	3029.2 (2498.7,3696.8)	175895 (128002,236042)	2288.4 (1699.6,2991.8)	-24.5 (-45.5,0.7)
**High-**	1527893	766.7	1038269	253	-67 (-

**income Asia Pacific**	(1442344,1607196)	(721.3,807.2)	(916660,1124028)	(232.8,271.5)	68.9,-64.9)
**Brunei Darussalam**	1657 (1405,1953)	1348.3 (1141.3,1576.1)	2287 (1947,2699)	600.9 (519.1,698.1)	-55.4 (-64.5,-42.8)
**Japan**	848224 (799737,884988)	512.8 (482.3,535.5)	776369 (691099,840237)	251.8 (231.9,268.2)	-50.9 (-52.6,-49.2)
**Republic of Korea**	658794 (585423,719430)	2204.9 (1944.1,2405.2)	245744 (214675,283292)	278.6 (243.9,320.6)	-87.4 (-88.8,-84.9)
**Singapore**	19219 (18342,20032)	801.3 (764.4,836.5)	13868 (12728,14989)	165.7 (151.4,179.6)	-79.3 (-80.8,-77.8)
**High-income North America**	923420 (878464,956606)	274.4 (261.7,284)	1343437 (1250162,1412207)	221.2 (208.2,232.8)	-19.4 (-22.6,-16.6)
**Canada**	72417 (67895,76948)	225.5 (211.7,239.9)	90457 (82077,96911)	132.5 (121.3,141.5)	-41.3 (-45.6,-36.8)
**Greenland**	484 (414,552)	1305.4 (1128.7,1475.4)	396 (332,463)	573.8 (484.4,669.8)	-56 (-63.9,-47.7)
**United States of America**	850498 (806832,881553)	279.8 (265.9,289.7)	1252563 (1164768,1317343)	231.8 (218.1,244.1)	-17.2 (-20.7,-14.1)
**North Africa and Middle East**	3396227 (2947395,3912469)	1507.8 (1332.2,1667.2)	3051893 (2633083,3493646)	614.4 (529.3,701.5)	-59.2 (-65.1,-52.3)
**Afghanistan**	259201 (188075,338217)	3325.6 (2401.3,4369.4)	241355 (173077,326234)	1752.2 (1210.9,2400.5)	-47.3 (-60.8,-29.2)
**Algeria**	166213 (136330,198353)	1131.9 (896.5,1395.5)	180749 (126506,253357)	506.5 (357.4,713.2)	-55.3 (-66.8,-42.5)
**Bahrain**	2627 (2389,2860)	1232.9 (1116.7,1354.8)	4258 (3612,4981)	447.6 (381.7,521.6)	-63.7 (-69.8,-55.7)
**Egypt**	819058 (546263,1080870)	2104.9 (1374.6,2742)	516111 (339533,692517)	735.8 (477.3,992.1)	-65 (-73.4,-56.2)

**Iran (Islamic Republic of)**	179957 (159922,207710)	540.4 (480.4,594.2)	186754 (171075,203510)	230.6 (210.9,251.6)	-57.3 (-62.6,-51.1)
**Iraq**	208255 (176963,247595)	1940.1 (1620.3,2328.2)	297781 (228418,374238)	1086 (840.2,1345.9)	-44 (-58.3,-25.1)
**Jordan**	22662 (19463,26508)	1031.2 (862.1,1238.2)	29676 (24459,35227)	345.2 (280.6,412.1)	-66.5 (-73.7,-57.1)
**Kuwait**	3855 (3614,4128)	370.3 (343.3,399.3)	7483 (6280,8910)	202.4 (169.5,240.1)	-45.3 (-54.7,-35)
**Lebanon**	31355 (24540,39509)	1374.7 (1075.8,1719.9)	19417 (16415,23159)	319.4 (270.2,382.6)	-76.8 (-82.5,-68.9)
**Libya**	25066 (20027,30557)	804.4 (628.7,1014.6)	33845 (24218,47236)	574.7 (408,800.4)	-28.6 (-46.7,-5)
**Morocco**	239020 (182487,301351)	1340.7 (976.8,1741.7)	251011 (172482,336757)	711.1 (494.6,949)	-47 (-60.4,-31.9)
**Oman**	11280 (8541,14345)	1151.2 (862,1505.3)	10353 (8120,12561)	392.4 (312.8,478.9)	-65.9 (-75,-51.3)
**Palestine**	16337 (13410,19496)	1258 (1003.7,1535.8)	16081 (13822,18321)	534 (458.7,614.5)	-57.6 (-66.6,-45)
**Qatar**	1847 (1533,2160)	1250.8 (1044.2,1467.3)	4312 (3347,5507)	329.8 (257,408.7)	-73.6 (-80.2,-65)
**Saudi Arabia**	123768 (99087,154152)	1485.7 (1148.8,1883.1)	206491 (160589,263007)	736.1 (597.1,890.6)	-50.5 (-63.6,-31.6)
**Sudan**	361634 (267788,467404)	2490 (1842.9,3084.8)	229271 (158311,319231)	896.2 (607.2,1231.6)	-64 (-75.3,-48.4)
**Syrian Arab Republic**	160940 (129213,197202)	1807.6 (1431.7,2200.8)	115702 (88144,150989)	864.2 (665.3,1110)	-52.2 (-65.8,-30.5)
**Tunisia**	49024 (39618,59771)	875.5 (688.3,1082.7)	55139 (35004,83689)	421.6 (266.5,638.5)	-51.8 (-67.7,-32.2)
**Turkey**	525484	1303.9	414909	451.6	-65.4 (-

	(444960,627196)	(1108.8,1515.2)	(344731,499256)	(375,538.7)	72.7,-56.3)
**United Arab Emirates**	6985 (5378,9031)	989.9 (754,1291.2)	18961 (14931,23110)	448.6 (370,536.1)	-54.7 (-65.6,-42.3)
**Yemen**	179801 (131368,231459)	2497.1 (1806.7,3293.4)	209389 (149622,286739)	1234.7 (858.4,1718.2)	-50.6 (-64.5,-30.1)
**Oceania**	109296 (86462,136691)	3385.9 (2700.3,4201.4)	211111 (167044,258871)	2582.5 (2058.7,3157.5)	-23.7 (-39.4,-3.7)
**American Samoa**	572 (505,641)	2256.4 (2014.1,2504)	818 (687,972)	1674.5 (1413,1989.4)	-25.8 (-38.7,-8.2)
**Cook Islands**	250 (211,292)	1902.2 (1626.3,2196.3)	220 (178,266)	895.1 (726.5,1080.3)	-52.9 (-63.5,-40.7)
**Fiji**	10587 (9076,12389)	2583.8 (2221.1,2986.1)	14050 (10868,17756)	1792.7 (1404.4,2237.4)	-30.6 (-46.1,-8.2)
**Guam**	906 (822,995)	1080.4 (984.8,1177.4)	1410 (1255,1589)	705.9 (630.5,792.7)	-34.7 (-43.6,-23.6)
**Kiribati**	1653 (1370,1947)	3843.2 (3164.2,4492.8)	2705 (2134,3421)	3214 (2579.2,3970.2)	-16.4 (-34,7.5)
**Marshall Islands**	791 (665,925)	4261.6 (3569.1,4984.7)	1391 (1055,1824)	3450.4 (2651.9,4450.8)	-19 (-36.2,3.3)
**Micronesia (Federated States of)**	2432 (1929,3021)	4557 (3658.3,5645.3)	2671 (2089,3433)	3276.4 (2591.7,4145.5)	-28.1 (-44.5,-3.1)
**Nauru**	267 (205,344)	4745.9 (3726.7,6028.8)	283 (216,371)	3998.5 (3121.7,5096.9)	-15.7 (-34.3,11.8)
**Niue**	57 (47,70)	2637.7 (2165.9,3241.7)	40 (33,48)	1922 (1574.8,2318.8)	-27.1 (-42.6,-8.9)
**Northern Mariana Islands**	500 (387,635)	2195.2 (1828.7,2688.6)	720 (623,793)	1365 (1186,1500.2)	-37.8 (-51.2,-23.3)
**Palau**	304 (246,375)	2812.7 (2291.4,343	470 (383,577)	2023.7 (1664.4,245	-28.1 (-45.2,-

		8)		5.2)	5.3)
**Papua New Guinea**	71442 (52622,94367)	3656.7 (2749,4778.8)	152418 (112896,198066)	2775.1 (2053.8,3536.3)	-24.1 (-45,6.4)
**Samoa**	2690 (2188,3197)	3009.8 (2465.3,3540.8)	3523 (2903,4323)	2337.2 (1938.2,2850.8)	-22.3 (-37.8,-0.2)
**Solomon Islands**	6155 (3949,7924)	4310.7 (3004.4,5404.2)	13946 (10862,17853)	3737.7 (2942.4,4694.8)	-13.3 (-34.8,22.7)
**Tokelau**	41 (33,51)	3086.8 (2423.8,3839.9)	27 (21,34)	1869.7 (1431,2353.7)	-39.4 (-52.5,-23.3)
**Tonga**	743 (597,893)	1292.7 (1048.8,1545.5)	831 (648,1032)	1015.9 (790.8,1257.4)	-21.4 (-41.6,4.2)
**Tuvalu**	320 (267,379)	4498.6 (3764,5303.4)	290 (240,352)	2688.2 (2232.3,3238.3)	-40.2 (-51.1,-25.7)
**Vanuatu**	2614 (2039,3279)	3644.5 (2927.7,4552.2)	5819 (4662,7136)	2943.3 (2389.8,3575.1)	-19.2 (-37.1,3)
**South Asia**	9251578 (7825574,10658981)	1428.6 (1209,1643.1)	15045582 (12776920,17158102)	972.4 (816.1,1110.7)	-31.9 (-41,-21.2)
**Bangladesh**	1543653 (1246174,1819400)	2780.7 (2250.3,3324.9)	2170499 (1682691,2773704)	1538.5 (1199.5,1949.4)	-44.7 (-57.3,-27.9)
**Bhutan**	3813 (2854,5015)	1335.8 (1004,1768.7)	4269 (3180,5470)	689.4 (518.3,874.5)	-48.4 (-63.4,-29)
**India**	6696449 (5545167,7835838)	1287 (1063.1,1507)	11098171 (9239866,12841993)	888.6 (733.2,1030.8)	-31 (-41.6,-18.8)
**Nepal**	174969 (139293,220599)	1628 (1275.4,2055.2)	225958 (172301,296343)	936.8 (713.4,1225.5)	-42.5 (-57.4,-22.3)
**Pakistan**	832694 (709614,974465)	1316.3 (1079.4,1560.2)	1546685 (1234501,1920048)	1111.5 (904.4,1389.8)	-15.6 (-32.7,7.6)
**Southeast Asia**	7928865 (7224788,8671143)	2801.3 (2529.2,3081.5)	13616701 (12011136,15407482)	1976.8 (1744.3,2238.7)	-29.4 (-38.6,-19.2)
**Cambodia**	182636	3630.7	289683	2294.7	-36.8 (-

	(153492,212778)	(3081.7,4226.4)	(221551,363150)	(1785.4,2850.9)	52,-18.4)
**Indonesia**	3588529 (3140224,4055052)	3196.1 (2753,3673.7)	6741746 (5461358,8249714)	2647.2 (2141.8,3241.4)	-17.2 (-32.1,-0.8)
**Lao People's Democratic Republic**	116273 (91583,149850)	4705.8 (3670.7,5956.4)	126434 (98017,159613)	2457.8 (1901.1,3073.5)	-47.8 (-61.9,-30)
**Malaysia**	205665 (187285,222660)	2036.2 (1841.8,2209.1)	338609 (308232,372901)	1152.6 (1045.3,1268.4)	-43.4 (-50.2,-35.8)
**Maldives**	3788 (3294,4531)	3547 (3090.9,4102.8)	3162 (2581,3754)	821.2 (670.4,974)	-76.8 (-82.1,-70.7)
**Mauritius**	15464 (14648,16424)	1926.8 (1826.7,2042.9)	13045 (12064,13735)	736.3 (680.8,774.3)	-61.8 (-65.1,-59.2)
**Myanmar**	1240141 (987903,1557848)	4722 (3784.7,5884.4)	1288648 (1035049,1629588)	2510.8 (2026.8,3159.6)	-46.8 (-60.6,-27.4)
**Philippines**	609768 (564647,656908)	1779.6 (1636,1927.1)	1542378 (1302134,1800681)	1696.1 (1435.7,1969.2)	-4.7 (-19.5,12.5)
**Seychelles**	949 (840,1090)	1672.2 (1483.5,1920.2)	1045 (902,1207)	859.1 (743.9,994.2)	-48.6 (-55.6,-40.6)
**Sri Lanka**	169288 (151140,188438)	1516 (1353.6,1685.1)	212752 (142521,290905)	805.4 (545.7,1089.6)	-46.9 (-65.3,-24.8)
**Thailand**	548831 (473958,621736)	1439.2 (1239.7,1632.3)	827878 (651226,1029518)	821.5 (649.4,1019.3)	-42.9 (-56.7,-24.7)
**Timor-Leste**	10274 (8479,12410)	2853.4 (2300.6,3470.6)	20932 (15553,27236)	2311.1 (1709.6,2994.5)	-19 (-40.9,7.2)
**Viet Nam**	1225790 (965881,1549104)	2982.8 (2349.6,3788.7)	2191395 (1747215,2643822)	2159 (1736.3,2587.2)	-27.6 (-46.3,-4.2)
**Southern Latin America**	510248 (487713,534958)	1090.8 (1042.8,1143.9)	314123 (295955,332443)	372.1 (350.5,394)	-65.9 (-68.3,-63.5)
**Uruguay**	32046 (30298,33775)	866.8 (819.3,914.	17254 (15965,18479)	335.3 (311.8,359.	-61.3 (-64.5,-

		1)		1)	57.9)
**Argentina**	387069 (367988,409929)	1195.7 (1136.7,1267.2)	225483 (211364,238981)	415.3 (389.3,440.4)	-65.3 (-67.8,-62.5)
**Chile**	91109 (86776,95860)	866.8 (825.1,911.4)	71369 (66204,76532)	286.4 (266.5,307)	-67 (-69.4,-64.8)
**Southern Sub-Saharan Africa**	397780 (357659,432326)	1281.2 (1127.4,1404.7)	754889 (693688,820770)	1224.2 (1130.5,1326.8)	-4.5 (-14,7.7)
**Botswana**	14102 (10500,18244)	2337.4 (1770.2,2975.2)	16866 (12737,22513)	1089.7 (832,1432.4)	-53.4 (-66.7,-35.5)
**Eswatini**	7404 (5991,9153)	2365.1 (1902.7,2905.6)	12607 (8300,18352)	2028.6 (1363.1,2894.9)	-14.2 (-40.1,21.9)
**Lesotho**	16934 (13262,21142)	1966 (1538.8,2458.3)	33030 (24087,45246)	2876.6 (2120.4,3879.4)	46.3 (3.3,116.5)
**Namibia**	15970 (13175,18995)	2313.5 (1905.5,2788.1)	23665 (17263,30898)	1644 (1218.5,2109.1)	-28.9 (-48.2,-6.9)
**South Africa**	289024 (256558,317248)	1183.8 (1007.9,1322.4)	513344 (460157,559193)	1050.2 (942.1,1143.7)	-11.3 (-19.1,-1.2)
**Zimbabwe**	54345 (44791,64379)	1290 (1055.4,1527.4)	155377 (119000,199626)	2015.2 (1577.5,2534.2)	56.2 (18,112.9)
**Tropical Latin America**	1369874 (1334113,1402941)	1322.3 (1284.4,1358.1)	1172380 (1118584,1218666)	449.7 (428.7,467.4)	-66 (-67.7,-64.5)
**Brazil**	1342959 (1307798,1375283)	1326.5 (1287.6,1361.7)	1135060 (1081881,1181019)	446 (425,464.3)	-66.4 (-67.9,-64.9)
**Paraguay**	26916 (23740,30770)	1127.5 (992.6,1284.3)	37320 (28431,47156)	615.4 (467.5,775.8)	-45.4 (-59.6,-28.6)
**Western Europe**	2318883 (2211365,2408674)	419.7 (401.4,435.3)	1488382 (1347101,1577865)	161.2 (149.8,170.2)	-61.6 (-63.5,-59.8)
**Andorra**	116 (85,156)	204.3 (149.8,271.4)	164 (122,210)	108.2 (80.2,138.8)	-47 (-64.9,-25.9)

**Austria**	45627 (42946,48408)	399 (376.9,420.7)	22562 (20295,24453)	124.1 (112.2,134.2)	-68.9 (-71.2,-66.6)
**Belgium**	65135 (61389,68529)	447 (422.5,469.2)	39513 (34941,43057)	170.6 (154.7,184.7)	-61.8 (-64.7,-58.8)
**Cyprus**	5010 (4321,5756)	712.5 (610.4,818.1)	3907 (3293,4514)	201.9 (170.9,230.8)	-71.7 (-77.1,-64.8)
**Denmark**	33673 (31743,35579)	435 (412.9,459.5)	20960 (19226,22480)	178 (164.6,190.8)	-59.1 (-61.9,-55.9)
**Finland**	25561 (23505,27968)	373.2 (344.9,407.8)	20286 (18147,21938)	165.4 (151.2,178.1)	-55.7 (-60,-51.1)
**France**	287570 (269447,303651)	363.7 (342.4,383.4)	187821 (168461,204118)	133.1 (121.1,144.4)	-63.4 (-66.2,-60.5)
**Germany**	453575 (413575,501559)	378.2 (346.5,414.7)	266748 (240637,287154)	143.7 (131,154)	-62 (-66.3,-58)
**Greece**	136561 (129048,142707)	930.3 (880.4,971.2)	89144 (80288,95133)	379.1 (349.3,402.2)	-59.2 (-61.6,-56.7)
**Iceland**	875 (817,934)	308.8 (288.8,328.5)	670 (588,742)	115.8 (103,128)	-62.5 (-66.2,-58.5)
**Ireland**	13302 (12571,14029)	330.4 (312.5,347.8)	8101 (7266,8811)	103.4 (93.2,112.3)	-68.7 (-71.3,-66.1)
**Israel**	24710 (23313,26076)	510.4 (481.4,538.8)	20101 (18349,21850)	164.3 (150.8,177.6)	-67.8 (-70.7,-65.1)
**Italy**	364589 (345790,378825)	432 (411.3,448.2)	269662 (237623,289330)	186.4 (170.5,198.2)	-56.9 (-59.3,-54.6)
**Luxembourg**	3279 (3108,3442)	623.1 (590.8,654.1)	1773 (1591,1966)	165.8 (149.1,183.8)	-73.4 (-76.2,-70.6)
**Malta**	2204 (2076,2335)	522.7 (490.9,553.9)	1449 (1291,1599)	161.1 (145.4,177.4)	-69.2 (-72.2,-65.7)
**Monaco**	303 (235,365)	473.4 (374.7,565.	177 (141,220)	203.9 (159.3,254.	-56.9 (-68.7,-

		3)		9)	40.8)
**Netherlands**	64970 (60768,68520)	331.7 (310.8,349.2)	50324 (44960,54068)	143.2 (129.8,153.8)	-56.8 (-60,-53.7)
**Norway**	18783 (17865,19585)	281.7 (268.8,293.6)	12814 (11578,13739)	123.4 (112.4,132.3)	-56.2 (-58.8,-53.8)
**Portugal**	163224 (155740,169985)	1211.3 (1155.4,1261.1)	64204 (58521,68889)	275.9 (254.2,295.1)	-77.2 (-78.7,-75.6)
**San Marino**	115 (97,134)	335.3 (282.2,389.8)	90 (63,124)	125.1 (86.6,172)	-62.7 (-74.9,-46.8)
**Spain**	276581 (259630,289188)	534.4 (503.3,558.3)	158737 (140787,172071)	162.5 (148.5,174.6)	-69.6 (-71.7,-67.5)
**Sweden**	42457 (39970,45031)	289 (273.2,305)	30434 (26712,33779)	138.2 (122.5,153.3)	-52.2 (-56.8,-47.2)
**Switzerland**	28219 (25719,30682)	277.6 (254.1,300.2)	15772 (13805,17362)	84.5 (75.2,92.3)	-69.6 (-72.8,-66)
**United Kingdom**	260539 (251130,267356)	304 (293.8,312)	201657 (186833,212286)	157.4 (148,165.4)	-48.2 (-50.1,-46.5)
**Western Sub-Saharan Africa**	2247400 (1806018,2728916)	2178.9 (1768.9,2637.1)	3223357 (2546461,3936778)	1375.3 (1095,1646.3)	-36.9 (-46,-25.4)
**Benin**	54335 (44579,65349)	2434.8 (2020.2,2956.5)	93795 (71075,115992)	1616.3 (1248.3,1978.5)	-33.6 (-47.8,-13.7)
**Burkina Faso**	86775 (66985,108470)	1731.1 (1373.4,2122.6)	148575 (109435,187639)	1384.7 (1035.6,1750.4)	-20 (-37.9,4.4)
**Cabo Verde**	2954 (2475,3436)	1242.8 (1024.1,1461.7)	4198 (3277,5268)	897.7 (705.6,1124.1)	-27.8 (-45.1,-4.8)
**Cameroon**	118773 (90115,156713)	2275.4 (1727.4,2994.4)	276017 (189171,380907)	1833.9 (1277.5,2524.9)	-19.4 (-40.1,11.7)
**Chad**	70429 (56813,85851)	2116.4 (1693.6,2560.3)	151884 (111464,201773)	2022.1 (1490.8,2678.5)	-4.5 (-28.7,24.3)

**Coted'Ivoire**	110497 (84466,147511)	2010.9 (1569.6,2616)	217568 (155228,286404)	1554.3 (1128.8,2029.1)	-22.7 (-42.5,5.1)
**Gambia**	8996 (6777,11515)	2074 (1583.7,2657.7)	21238 (16361,26960)	1890.2 (1449.3,2412)	-8.9 (-32,25.4)
**Ghana**	207748 (165744,258491)	2709.3 (2152.9,3403.3)	382247 (285721,486121)	1980.9 (1464.7,2544.9)	-26.9 (-45.2,0.3)
**Guinea**	90681 (70544,114149)	2295.5 (1838.7,2854)	127742 (92467,165084)	1900.3 (1395.8,2455.1)	-17.2 (-38.7,13.1)
**Guinea-Bissau**	19752 (14953,26409)	3957.4 (3035.8,5223.2)	25872 (19420,33090)	2867.1 (2153,3666)	-27.6 (-46.3,-5)
**Liberia**	33099 (25036,42820)	2217.4 (1765.4,2734.8)	45497 (32283,61766)	1729.9 (1272.9,2306.3)	-22 (-42.6,6.4)
**Mali**	107658 (82141,137091)	2194.8 (1676.8,2752)	161748 (119932,207185)	1464.6 (1083.8,1877)	-33.3 (-47.6,-13.7)
**Mauritania**	27638 (22364,34670)	2507.2 (1998.7,3188.4)	31346 (23314,42689)	1333.7 (994.3,1813.9)	-46.8 (-60.3,-26.9)
**Niger**	89836 (63375,130382)	2303.7 (1694.4,3086.1)	174553 (117976,240970)	1780.4 (1234.4,2404)	-22.7 (-41.3,1.2)
**Nigeria**	1033623 (780139,1335482)	2091.5 (1595.9,2685.4)	1049149 (791436,1372018)	966.2 (743.2,1239.1)	-53.8 (-63.9,-37.7)
**Sao Tome and Principe**	1184 (991,1387)	1667.1 (1403.8,1947.2)	1755 (1408,2227)	1368.5 (1124.2,1700.5)	-17.9 (-35.1,6.1)
**Senegal**	87109 (70853,108501)	2176.7 (1793.6,2675.3)	138317 (104373,175927)	1601.3 (1206.5,2042.9)	-26.4 (-43.8,-5.2)
**Sierra Leone**	60441 (45950,79450)	2224.7 (1782.6,2808.3)	85990 (62276,116334)	1816.8 (1344,2406)	-18.3 (-36.9,8.1)
**Togo**	35798 (28928,43208)	2254.3 (1864.5,2731.9)	85828 (60621,113149)	1930.6 (1411.2,2518.3)	-14.4 (-36.7,13.3)

95% UI=95% uncertainty intervals. ASR: Age-standardized rate

Supplementary Figure1: Age-standardised disability adjusted life year (DALY) rates of intracerebral hemorrhage for the 203 countries by sociodemographic index, 1990–2021. Thirty-two points are plotted for each region and show the observed age standardised DALY rates from 1990 to 2021 for that region. Expected values, based on sociodemographic index and disease rates in all locations, are shown as a solid line. Regions above the solid line represent a higher than expected burden.

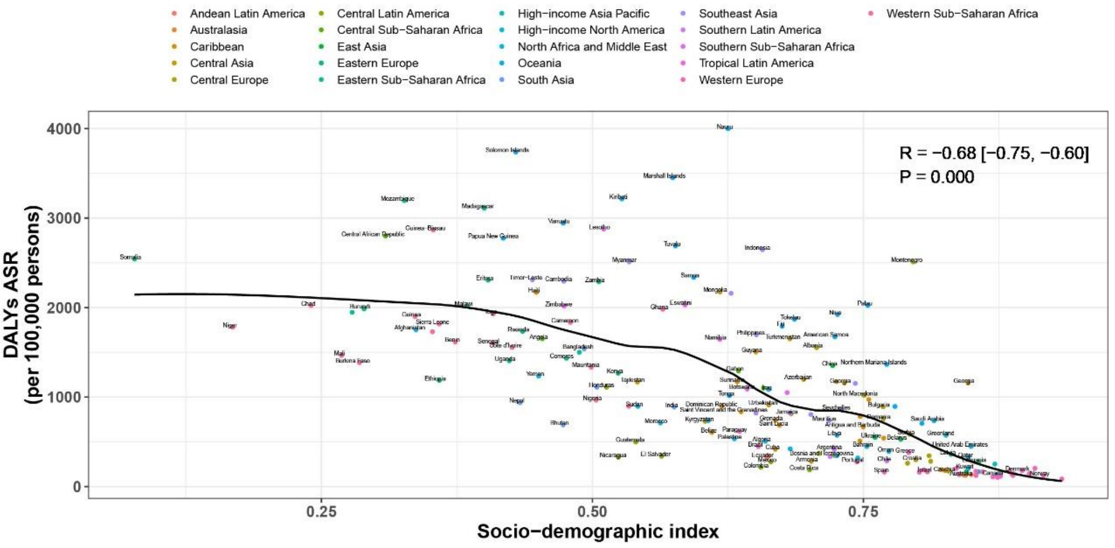

Supplementary Figure2: The percentage of disability-adjusted life years (DALYs) due to intracerebral hemorrhage attributable to each risk factor for males in the 21 Global Burden of Disease regions in 2021.

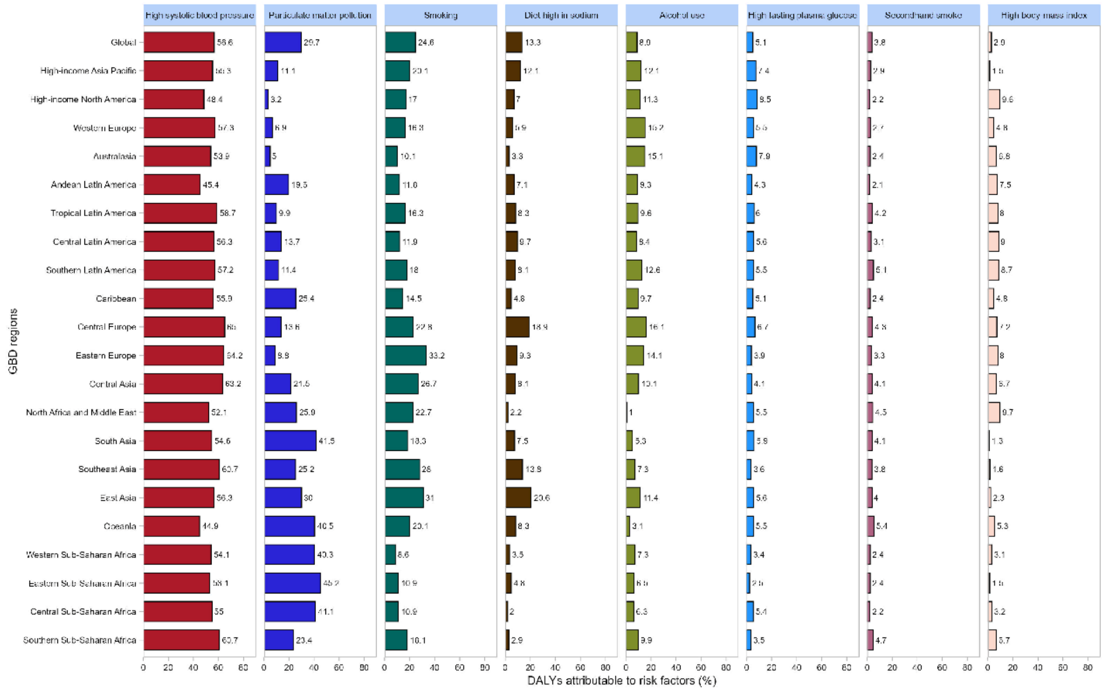

Supplementary Figure3: The percentage of disability-adjusted life years (DALYs) due to intracerebral hemorrhage attributable to each risk factor for females in the 21 Global Burden of Disease regions in 2021.

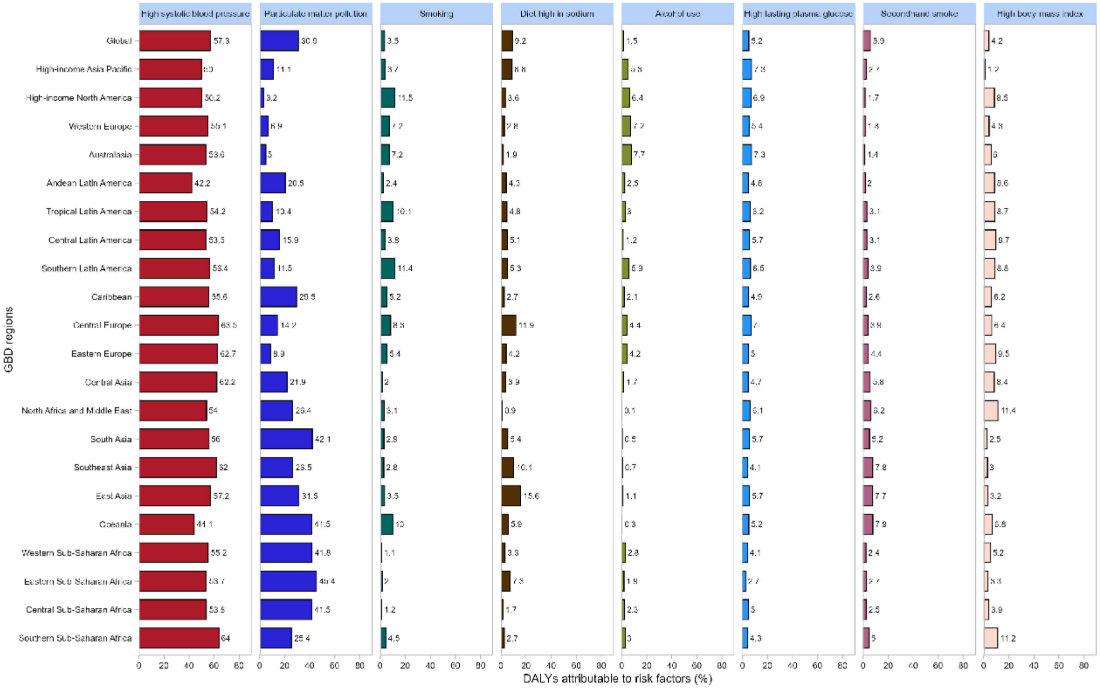

Supplement: Supplementary file 1 — Supplementary Material 1 [file 12889_2024_19923_MOESM1_ESM.pdf]
